# Uncertainty Analysis of a Multi-Sensor Fusion Measurement Chain for Blade Collision Warning in Coaxial Twin-Rotor Helicopters

**DOI:** 10.3390/s26175426

**Published:** 2026-08-27

**Authors:** Wenjie Zheng, Zhen Qiu, Zewen Dong, Wenchuan Hu, Yongqiang Qiu, Zurong Qiu

**Affiliations:** 1 State Key Laboratory of Precision Measuring Technology and Instruments, Tianjin University, Tianjin 300072, China; tjbyzwj@163.com (W.Z.); dongzenwen1995@163.com (Z.D.); 2 School of Engineering, University of Greater Manchester, Bolton BL3 5AB, UK; 3 College of Mechanical Engineering, Tianjin University of Technology and Education, Tianjin 300222, China; huwenchuan@tute.edu.cn; 4 School of Engineering, Liverpool John Moores University, Liverpool L3 3AF, UK; y.qiu@ljmu.ac.uk

**Keywords:** coaxial twin-rotor helicopter, blade collision warning parameter, multi-sensor fusion, correlated uncertainty propagation

## Abstract

A coaxial twin-rotor helicopter features a compact structure by eliminating the tail rotor. Although offering advantages in lift capability and maneuverability, the design presents challenges from its mechanical complexity and the aerodynamic interference between the counter-rotating rotors. During blade intersection, the collision risk of the blades depends not only on the blade-tip distance, but also on the intersection phase and the blade-tip position. In our study, we defined a blade collision warning parameter, *d*, to represent a fused safety clearance in coaxial twin-rotor helicopters, and proposed a correlated uncertainty propagation model for the measurement chain. The proposed model incorporates uncertainty sources from radar ranging, phase determination, geometric consistency, phase-synchronized triggering, sensor-point substitution, and model discrepancy through covariance terms. Experimental validation is performed on a single-rotor blade-intersection platform under controlled conditions. With the simulated blade rotated at 420 r/min, the combined standard uncertainty ranges from 0.677 to 0.996 mm over the reference warning parameter range of 99–990 mm. The event-level residual-compatibility rate is 86.8%, with localized non-compatibility observed at several reference points. Additional tests at 300 and 600 r/min demonstrated millimeter-level stability. Our uncertainty analysis identified radar ranging as the dominant contributor, followed by model discrepancy and sensor-point substitution uncertainty.

## 1. Introduction

Coaxial twin-rotor helicopters operate without a tail rotor and use a configuration in which the upper and lower rotors rotate coaxially in opposite directions. This configuration provides substantial advantages in structural compactness, lift utilization, hovering efficiency, low-speed maneuverability, and the takeoff and landing capability in confined spaces. As a result, coaxial twin-rotor helicopters have considerable application potential in shipborne aircraft, unmanned helicopters, urban low-altitude flight platforms, and heavy-lift vertical takeoff and landing systems [1,2,3,4]. However, this configuration also introduces a safety issue that does not exist in conventional single-rotor systems: the upper and lower rotors rotate at high speed in opposite directions about the same axis, and the spatial clearance between blades is jointly affected by blade flapping, lead–lag motion, torsion, elastic deformation, aerodynamic interference, and changes in flight state. As the upper and lower blades approach at the intersection phase, reliable real-time collision-risk information is crucial; without such information, the structural safety assessment and warning decision-making cannot be effectively completed. Therefore, dynamic safety-clearance measurement during blade intersection is a critical issue in the safety monitoring of coaxial twin-rotor helicopters [5,6,7,8].

Characterizing the collision risk of coaxial twin-rotor blades is challenging, as it cannot be adequately represented by a single local distance measured at a specific instant and location. This is because blade intersection between a pair of upper and lower blades is not a static geometric proximity problem, but a dynamic spatial relationship that varies with rotor phase, blade attitude, and structural deformation. During an intersection event, not only the local blade-tip distance between the pair of the upper and lower blades changes, but also the blade tips’ relative position to the rotor axis, and the blade hazard boundary. In this case, the instantaneous measurement of the blade tips’ distance alone is not enough to determine the critical safety distance between the upper and lower blades, making it more difficult to convert the measurement into a unified risk metric for warning decision-making. Therefore, in order to comprehensively represent the degree of hazardous proximity between the upper and lower rotors, we introduced a blade collision warning parameter in this paper. This blade collision warning parameter will integrate the blade-intersection phase, blade-tip position, and blade-tip distance into a single-valued risk descriptor for safety assessment, under a unified spatial reference frame and a unified time base.

Extensive research has been conducted on rotor-blade motion characterization and inter-blade clearance measurement. Optical measurement, ultrasonic measurement, digital image correlation, video-based measurement, and structural state identification methods have been widely used to measure blade displacement, deformation, attitude, and load-related parameters [9,10,11]. The stereoscopic pattern recognition method used in the HART II (Helicopter Aeromechanics Research Test II) can obtain rotor-blade motion information, and Schneider systematically analyzed this class of optical rotor measurement methods, demonstrating their high application value in blade-motion reconstruction [12]. Kumar directly examined the relationship between HART II data and aeroelastic analysis, and analyzed the response correlations among flapping, lead–lag, and torsional modes [13]. Sirohi and Lawson used digital image correlation to measure rotor-blade deformation, providing an effective approach for obtaining noncontact blade deformation fields [14]. The video-based measurement system proposed by Wei et al. further extends the available methods for dynamic rotor-blade deformation measurement [15]. Uehara and Sirohi conducted full-field optical deformation measurements on flexible rotors, highlighting the advantages of vision-based measurement in analyzing the dynamic structural response of rotor blades [16]. These studies provide important technical support for rotor dynamics analysis, structural health monitoring, and aeroelastic modeling.

Recent studies further emphasize the importance of dynamic-state and uncertainty effects in rotating and kinematic measurement systems. Liu et al. reviewed the vibration behavior of flexible rotating wind-turbine blades and showed that blade responses are strongly affected by structural dynamics and operating conditions, highlighting the need to account for state-dependent deformation when extending measurements from a rigid platform to an actual rotating system [17]. From the measurement perspective, Ernst et al. experimentally evaluated the uncertainty of a kinematic LiDAR-based multi-sensor system and demonstrated the importance of assessing uncertainty at the fused system-output level rather than considering sensor accuracy alone [18]. These developments further motivate a system-level uncertainty treatment for the dynamic multi-sensor measurement chain considered here.

However, when the research objective shifts from blade-motion characterization to collision warning in coaxial twin-rotor helicopters, existing methods still have several limitations. Firstly, many optical and photogrammetric methods mainly output blade displacement fields, deformation fields, or attitude information. Although these outputs can describe the blade-motion states in detail, they do not provide a direct risk assessment that comprehensively reflects the blade status for collision warning. Secondly, these methods usually rely on specific camera arrangements and complex offline post-processing procedures, making it difficult to meet the requirements of in situ real-time measurement during high-speed blade intersection in coaxial rotors. Thirdly, existing studies focus more on the measurement accuracy of individual modules or local measurement results. Insufficient attention has been paid to identifying the dominant uncertainty sources and analyzing how multi-source measurements propagate through the measurement chain towards the ultimate safety assessment. In summary, existing methods are either difficult to deploy in situ and in real time to obtain a comprehensive collision-risk assessment or lack a system-level measurement uncertainty analysis.

To address the above issues, we previously proposed a phase–position–distance cooperative measurement system for blade collision warning in coaxial twin-rotor helicopters [19]. In that system, an encoder obtains the blade-intersection phase and determines the valid intersection instant and triggering window; a tracking camera measures the blade-tip position in a unified reference coordinate system to determine the intersection geometry; and a millimeter-wave radar measures the instantaneous distance between the upper and lower blade tips within the intersection time window. The measured phase, position, and distance information are then fused to reconstruct a warning parameter for blade collision. This method maps multi-source measurements into a unified parameter that reflects the safety margin, thereby better meeting the practical requirements for collision warning in coaxial twin-rotor helicopters.

The previous study demonstrates the feasibility of dynamically measuring the blade collision warning parameter to assess the blade collision risks. In the phase–position–distance fusion measurement chain, the encoder, tracking camera, and millimeter-wave radar are not independent measurement units. Therefore, evaluating the accuracy of the individual sensor modules alone is insufficient to establish a reliable warning parameter. The camera and radar share the phase-triggering chain, while the measured phase, blade-tip position, and inter-blade distance are coupled through geometric calibration parameters and coordinate-registration relationships. Consequently, the uncertainties associated with these input quantities do not propagate independently to the fused output. Since the warning decision is based on comparing the final fused parameter with a predefined safety threshold, the uncertainty evaluation must be performed at the level of the final measurand rather than inferred from the nominal accuracies of the individual sensors. To this end, in this study we establish a system-level correlated uncertainty propagation model for the phase–position–distance fusion measurement chain and validate the model experimentally on a blade rotating simulation platform.

In the following sections, Section 2 defines the blade collision warning parameter and develops the general and reduced measurement models. Section 3 establishes the correlated uncertainty propagation framework. Section 4 describes the test platform, experimental conditions, and procedures used to evaluate the effective-input uncertainties and covariance matrices. Section 5 presents and discusses the results of propagated uncertainty, repeatability residual compatibility, uncertainty contributions, and system robustness. Finally, Section 6 summarizes the conclusions and discussion on extending the framework to a complete coaxial twin-rotor system.

## 2. Warning Parameter and Measurement Model

### 2.1. Warning Parameter Definition

To provide a consistent description of the degree of hazardous proximity during blade intersection in a coaxial twin-rotor helicopter, this study uses the blade collision warning parameter, d, as the final measurand. This parameter is neither a standalone measurement of the local distance nor the axial height difference associated with a single blade-tip point. Instead, it is a fused parameter jointly determined by the intersection phase, blade-tip position, and blade-tip distance. This blade collision warning parameter d represents the safety clearance formed by the hazardous boundaries of the upper and lower rotors in a unified spatial reference frame. A larger value of d indicates a larger safety margin between these boundaries, whereas a smaller value of d indicates a higher risk of hazardous proximity between the upper and lower blades.

**Figure 1:**
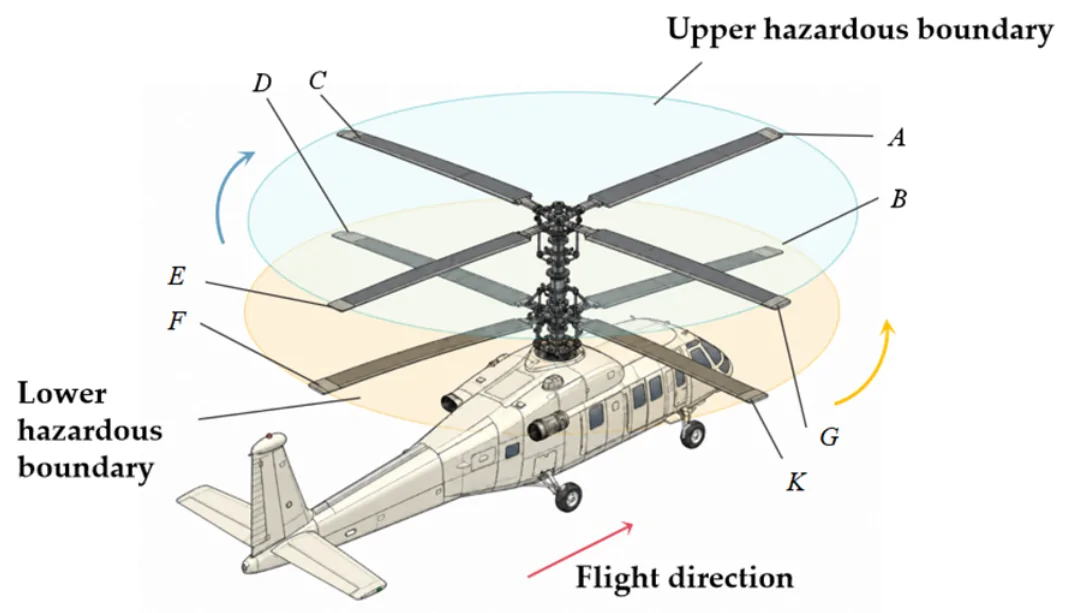
Schematic diagram of the hazardous boundaries for an eight-blade, coaxial twin-rotor helicopter. A, C, E, and G, as well as B, D, F, and K, are the blade-tip observation points for the upper and lower rotors, respectively.

Figure 1 illustrates an eight-blade coaxial twin-rotor system as an example. Within one 360° rotor revolution, the upper and lower blades intersect at fixed phase intervals. The four blade-tip observation points of the upper rotor are denoted as A, C, E, and G, and the four blade-tip observation points of the lower rotor are denoted as B, D, F, and K. At a given intersection state, the upper blade-tip point with the lowest axial position and the lower blade-tip point with the highest axial position may belong to different intersecting blade pairs. As a result, the local blade-tip distance measured between any blade pair does not necessarily represent the critical safety clearance. The collision risk should therefore be assessed using the concept of a hazardous boundary, which is defined by the boundary points corresponding to the extreme blade-tip positions of the upper and lower rotors in the intersection state. The minimum safety margin of the hazardous boundary under critical operating conditions is determined by the safety design and control response constraints of the specific helicopter.

The fuselage coordinate system can be defined as O-XYZ, where the OZ axis is aligned with the axis of the coaxial rotors. The axial coordinates of the four blade-tip observation points of the upper rotor in this coordinate system are denoted as $Z_{A}$, $Z_{C}$, $Z_{E}$, and $Z_{G}$, respectively. Similarly, the axial coordinates of the four blade-tip observation points of the lower rotor are denoted as $Z_{B}$, $Z_{D}$, $Z_{F}$, and $Z_{K}$, respectively. In the current intersection state, the hazardous boundary point of the upper rotor is determined as the blade-tip observation point with the lowest axial position among the upper rotor blade-tip observation points, namely,

(1) $$Z_{u}=\min(Z_{A},Z_{C},Z_{E},Z_{G}),$$

The hazardous boundary point of the lower rotor is determined as the blade-tip observation point with the highest axial position among the lower-rotor blade-tip observation points, namely,

(2) $$Z_{l}=\max(Z_{B},Z_{D},Z_{F},Z_{K}),$$

**Figure 2:**
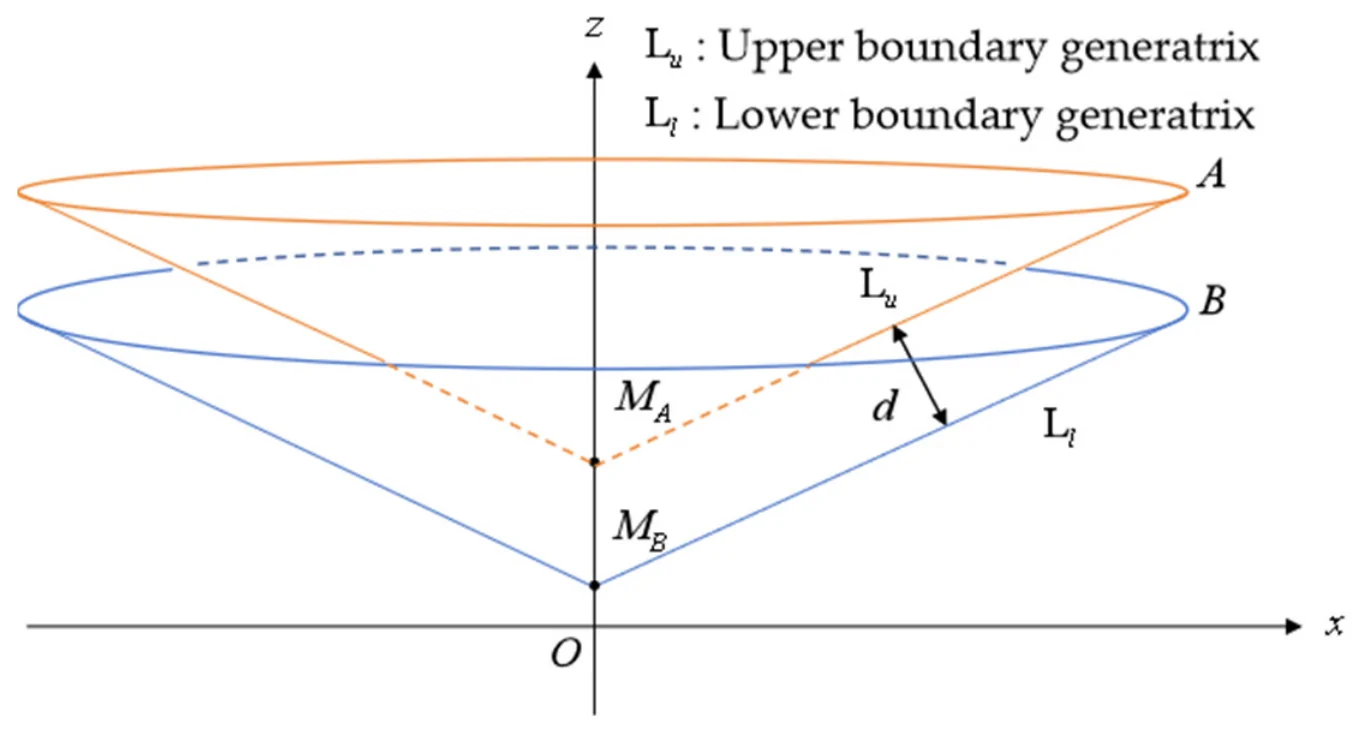
Construction of the upper and lower boundary cones. A and B are the blade-tip observation points for the upper and lower rotors, respectively.

Figure 2 illustrates a simplified representation of a specific case for one blade pair. In this case, the blade-tip observation points A and B are the hazardous boundary points of the upper and lower rotors, respectively. $M_{A}$ and $M_{B}$ are the centers of the two blade-roots, respectively. The upper and lower hazardous boundary cones can be constructed using $M_{A}A$ and $M_{B}B$. Let k be the slope of the generatrix of the upper cone; then, the normal distance between the upper and lower hazardous boundary cones can be defined as the blade collision warning parameter d. It is a calculation based on fused measurements and system parameters. The uncertainties associated with these input quantities and their propagation to d are formulated explicitly in Section 3.

### 2.2. General Fusion Measurement Model

With the blade collision warning parameter defined, the calculation of this parameter d requires three measurements and a set of system geometric and timing parameters.

The first measurement is the intersection phase, denoted as θ in this paper. Phase information is provided by the angular encoders and is used not only to determine whether the upper and lower blades satisfy the predefined intersection condition, but also to provide a timing reference. The second measurement is the spatial position of the blade tip, denoted as p. In the proposed measurement chain, the blade-tip position is obtained from the tracking cameras [20]. The third measurement is the local distance between the upper and lower blade-tip observation points, named as the blade-tip distance and denoted as h. It is obtained by the millimeter-wave radar, which has better environmental adaptability when compared with ultrasonic ranging sensors [21,22,23]. Together with θ and p, h can be mapped to create the parameter d.

As mentioned previously, the upper and lower rotors rotate in opposite directions in a complete coaxial twin-rotor system. Each blade may exhibit different flapping, lead–lag, torsional, and elastic deformation states at different intersection phases. Therefore, when calculating the warning parameter, the spatial positions should be considered for multiple upper and lower blade-tip observation points, as well as the selection of the intersecting blade pairs.

The positions of the four upper-rotor blade-tip observation points in the fuselage coordinate system are defined as

(3) $$P_{u,i}={\left[X_{u,i},Y_{u,i},Z_{u,i}\right]}^{T},i=1,2,3,4,$$

The positions of the four lower-rotor blade-tip observation points are defined as

(4) $$P_{l,j}={\left[X_{l,j},Y_{l,j},Z_{l,j}\right]}^{T},j=1,2,3,4,$$

where i and j are the indices of the blade-tip observation points for upper and lower blades, respectively. In practical measurements, the positions of the upper blade-tip observation points can be obtained using the tracking camera. The positions of the lower blade-tip observation points can then be constructed from the upper blade-tip positions $P_{u,i}$, the corresponding blade-tip distances $h_{ij}$, and the intersection phase θ. For an intersecting blade pair $\left(i,j\right)$, the lower blade-tip observation point is expressed as

(5) $$P_{l,j}=P_{u,i}+h_{ij}n_{ij}\left(\theta \right),$$

where $n_{ij}$ is the unit direction vector from the upper blade-tip observation point to the corresponding lower blade-tip observation point at the intersection phase θ.

After the axial coordinates of all upper and lower blade-tip observation points are obtained, the upper and lower boundary points are determined by Equations (1) and (2), respectively. We use I and J to denote the indices of the determined upper and lower boundary points in the current intersection state, respectively. The warning parameter for the current intersection state can then be rewritten as

(6) $$d=\frac{Z_{u,I}-Z_{l,J}}{\sqrt{1+k^{2}}},$$

Equation (6) reveals an important feature when calculating the warning parameter in a coaxial twin-rotor system: the determined upper and lower boundary points might not belong to the same pair of intersecting rotor blades. The uncertainty propagation in a complete coaxial twin-rotor scenario exhibits a piecewise nature and is related to the determined pair of boundary points. If the axial coordinates of two candidates of the boundary points are very close to each other, even a small environmental disturbance and measurement error can alter the result of the boundary point selection, causing boundary-point selection switching. In such a case, the calculation of d and the Jacobian matrix will change accordingly. Therefore, for a real coaxial twin-rotor system, uncertainty analysis must consider the stability of the boundary point selection.

In addition to the three primary measurements described above, calculation of d also depends on a set of system geometric and timing parameters. System geometric parameters, categorized as the vector η, include the coordinate transformation between the fuselage coordinate system, the rotor coordinate system, and the sensor coordinate system, the sensor installation, and the blade geometry. The timing parameters, including the triggering delay, sampling delay, and inter-module time-synchronization parameters, are grouped as the vector τ. Accordingly, the general fusion measurement model of d can be written in an abstract form as

(7) $$d=f\left(\theta ,p,h,\eta ,\tau \right),$$

### 2.3. Reduced Fixed-Pair Measurement Model

**Figure 3:**
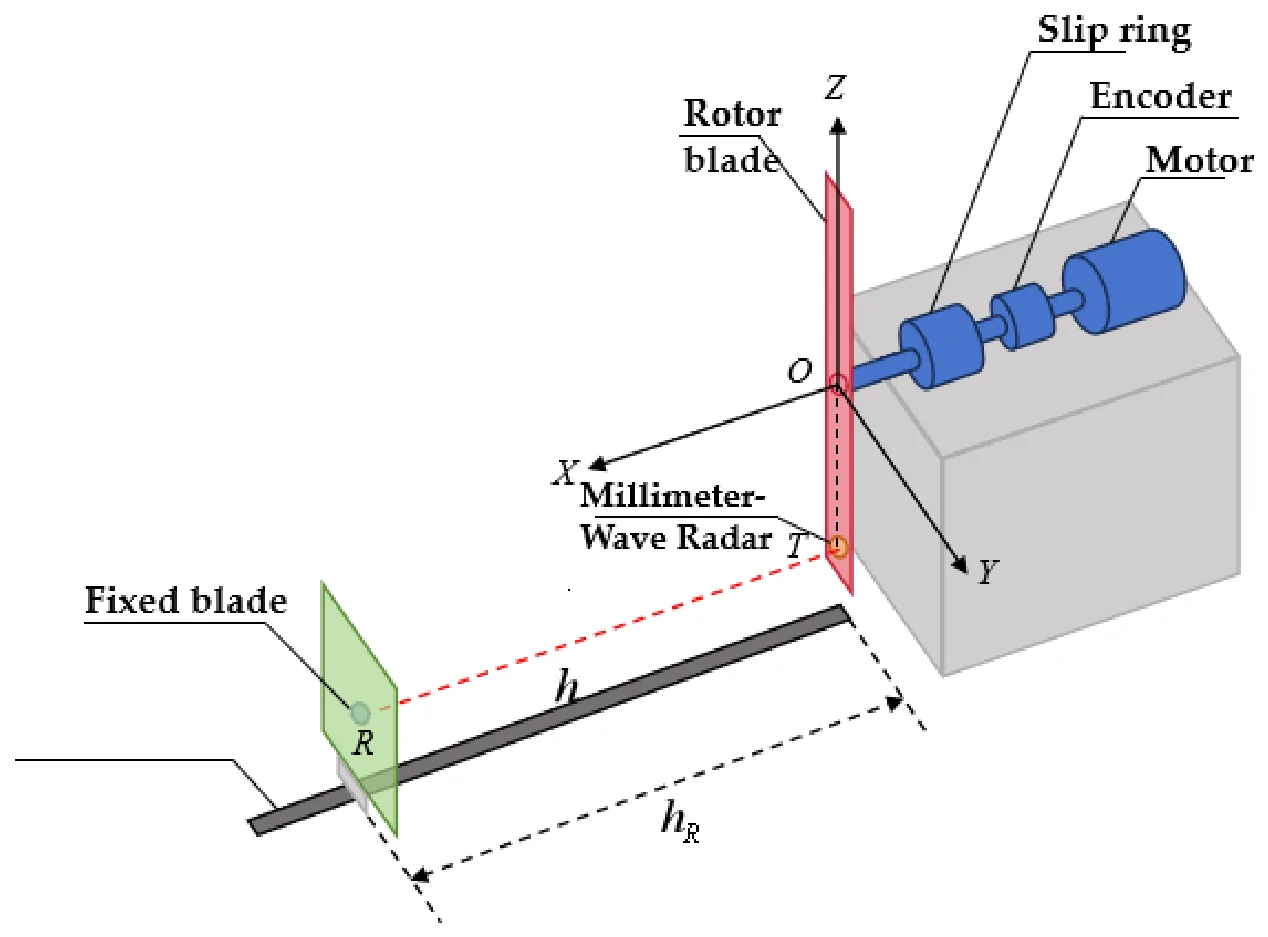
Design diagram of the test platform for experimental validation.

**Figure 4:**
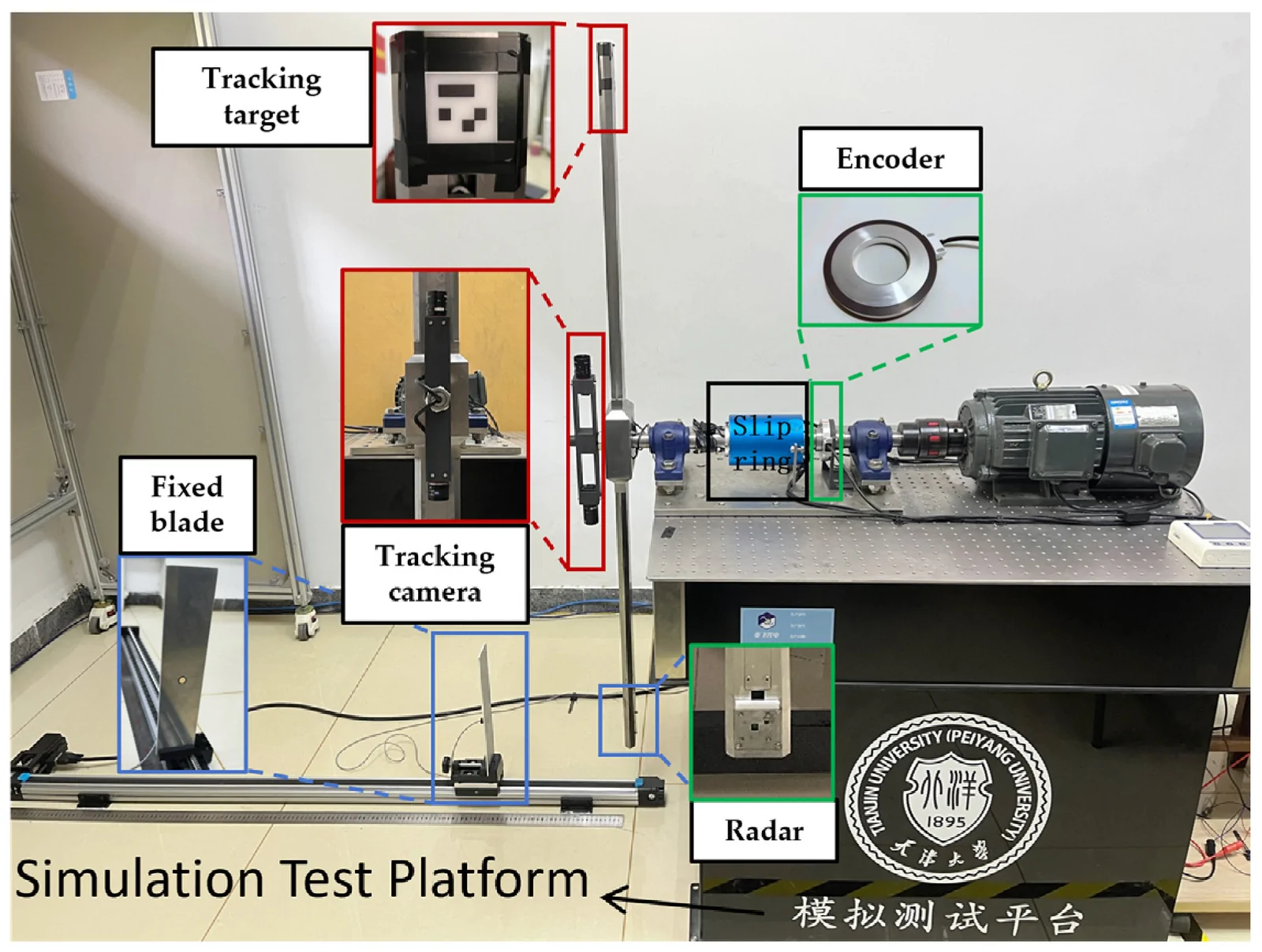
Photo of the test platform with key components.

The experimental validation in this study is based on a single-rotor blade-intersection platform. The design diagram of the platform is shown in Figure 3, and the platform photo can be found in Figure 4. The platform has one simulated blade stationed at the nominal intersection position, and another blade driven by a motor to rotate and sweep through the measurement region. The relative distance between the two simulated blade tips can be adjusted using a linear guide rail, allowing the reference blade-tip distance to be varied over the tested range. $h_{R}$ in Figure 3 denotes the reference local blade-tip distance set by the guide rail. An angular encoder (ITD69H00, Baumer, Frauenfeld, Switzerland) provides the phase reference, a rotor-mounted co-rotating camera (A7900MG13, Huarui Technology, Hangzhou, China) measures the rotating-side blade-tip position, and a millimeter-wave radar (TRA_120_045, Silicon Radar GmbH, Frankfurt (Oder), Germany) measures the local inter-blade distance at the intersection.

The present platform provides a controlled validation environment for the key measurement chain. Both blades are made of rigid materials and do not exhibit flapping or lead–lag motion. By fixing one blade at the intersection position and rotating the other through the sensing region, the platform simplifies the complete coaxial twin-rotor system to a fixed intersecting blade pair for experiments. The simplified platform reproduces the essential conditions required for measurement-chain validation, including deterministic intersection phase, controllable blade-tip distance, short-duration intersection events, and synchronized acquisition of phase, position, and distance information. Therefore, although the platform simplifies blade flexibility, counter rotation, and aerodynamic coupling, it remains functionally meaningful for evaluating phase-triggered acquisition, multi-sensor fusion, and warning parameter construction under dynamic blade-intersection conditions.

**Figure 5:**
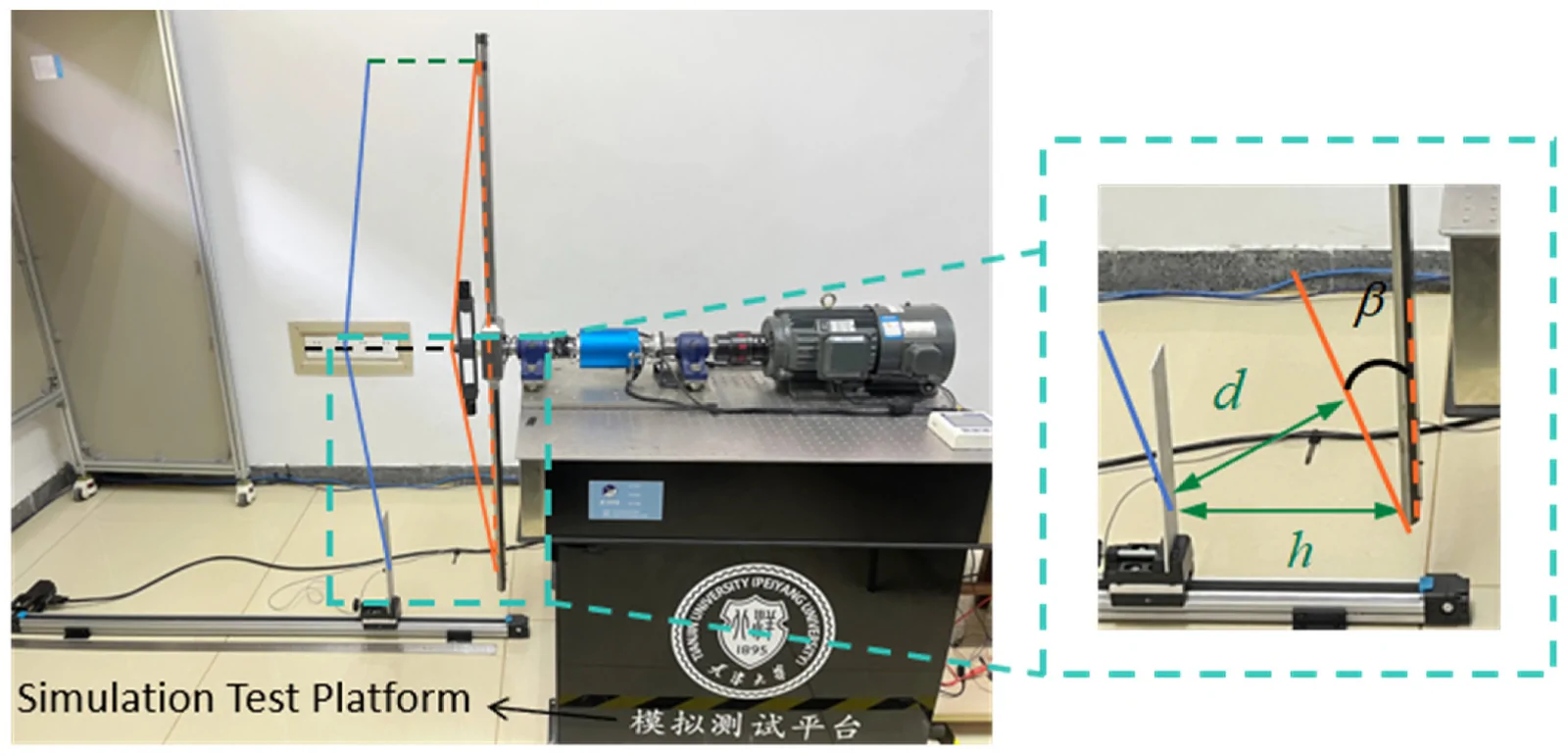
Measurement geometry on the test platform, illustrating the local inter-blade distance h, construction angle β, and corresponding blade collision warning parameter d.

Marked in Figure 5, the millimeter-wave radar measures the local distance h between the rotating blade tip and the fixed blade tip, whereas the tracking camera captures the position of the rotating blade tip. The construction angle β is the base angle of the cone formed by the rotating blade. It represents the geometric conversion relationship between h and d. When the intersecting blade pair is fixed and the construction angle is small, the warning parameter is mainly determined by the radar-measured distance h, with a geometric correction introduced by the construction angle β.

Under the stated test platform assumptions, the nominal platform measurement model is reduced to

(8) d=hcosβ,

Equation (8) does not replace the complete coaxial twin-rotor model in Equation (6). It represents the conditional form of the measurand when the boundary-point pair is fixed and the platform geometry suppresses blade deformation and selection switching. The uncertainty evaluated experimentally in this study therefore applies directly to the reduced platform model, whereas extension to a complete coaxial twin-rotor system additionally requires treatment of boundary-point selection switching and state-dependent deformation.

The construction angle β is determined by the geometric relationship between the rotating blade-tip observation point and rotating blade-root center point M. From Equation (5), the position of the rotating blade-tip observation point is defined as $P\left(X,Y,Z\right)$, and the position of rotating blade-root center as $P_{M}\left(X_{M},Y_{M},Z_{M}\right)$.

The radial projection distance between the rotating blade-tip observation points and rotating blade-root center point is then defined as

(9) $$\rho =\sqrt{{\left(X-X_{M}\right)}^{2}+{\left(Y-Y_{M}\right)}^{2}},$$

and the axial height difference is defined as

(10) $$q=Z-Z_{M}.$$

Accordingly, the construction angle can be written as

(11) $$\beta =\arctan\left(\frac{q}{\rho }\right).$$

When considering the mapping of the radar-measured local distance to the warning parameter under the experimental platform conditions, Equation (8) shows that the uncertainty of the position measurement mainly comes from the construction angle β, whereas distance measurement deviation enters d directly through h. To determine how measurement uncertainties affect the warning parameter, this mapping relationship must be linearized. The corresponding first-order propagation model is established in Section 3. When β is small, cosβ≈1, and the distance measurement error is therefore propagated almost proportionally to the final warning parameter. By contrast, the effect of position measurement error on d is weighted by the factor hsinβ.

The following section uses the reduced model from the experimental platform to establish a correlated uncertainty propagation framework under controlled conditions and then discusses its extension to complete coaxial twin-rotor systems involving the switching of boundary point selections.

## 3. Correlated Uncertainty Propagation

This section develops the correlated uncertainty model based on the reduced fixed-pair measurement equation. The formulation includes effective input deviations, timing and geometric coupling, first-order covariance propagation, covariance-allocated contributions, and a theoretical extension to boundary-point switching in the full rotor system.

### 3.1. Effective Input Deviations and Correlations

Under the fixed-intersection-pair and rigid-blade conditions defined in Section 2.3, the nominal blade collision warning parameter is expressed as

(12) $$d_{0}=h_{0}\cos\beta_{0},$$

where $h_{0}$ denotes the nominal local blade-tip distance at the theoretical intersection state and $\beta_{0}$ is the corresponding nominal construction angle.

In a practical measurement chain, the nominal model is affected by residual ranging deviation, vision-based angular deviation, inter-module geometric misalignment, timing deviation, and discrepancies between the sensor-observed points and the ideal geometric observation points. After deterministic calibration corrections are applied, the residual measurement model is expressed as

(13) $$d=h\cos\left(\beta +\lambda \right)+\Delta d_{sub}+\Delta d_{res},$$

where λ denotes the residual signed in-plane angular misalignment between the radar ranging direction and the nominal inter-blade distance direction in the construction plane. The nominal value of λ is zero after geometric registration. The nominal radar ranging direction is obtained from the calibrated radar installation geometry, while the nominal inter-blade distance direction is constructed from the calibrated blade-tip geometry. Their signed angular difference projected onto the construction plane defines λ; the mean offset is corrected during registration, and the standard uncertainty of the remaining Δλ is evaluated from repeated geometric registration.

The term $\Delta d_{sub}$ denotes the residual sensor-point substitution component after the deterministic point-location corrections are applied. For the radar branch, the effective electromagnetic scattering center does not necessarily coincide with the geometrically defined blade-tip reference point. Its mean offset is therefore identified independently by near-field dominant-scattering-center reconstruction, and treated as a deterministic point-location correction. Only the residual uncertainty associated with the reconstructed scattering-center location is propagated through $\Delta d_{sub}$. For the vision branch, the corresponding target-to-tip substitution is determined from the calibrated rigid geometry between the tracking target and the blade-tip reference point. The residual radar range-direction deviation is not included again in $\Delta d_{sub}$, because it is already represented by the ranging input Δh. The term $\Delta d_{res}$ denotes the residual model-closure discrepancy with all explicitly modeled measurement deviations and identifiable deterministic corrections accounted for. Under the present rigid fixed-pair platform condition, it represents the remaining difference between an independently calibrated geometric representation of the blade-intersection state and the reduced mapping d=hcosβ.

Both $\Delta d_{sub}$ and $\Delta d_{res}$ are treated as independently evaluated input quantities. When their deterministic components can be identified, these components should be corrected first. Only the residual components and the uncertainties associated with the applied corrections are propagated through Equation (13).

The effective input-deviation vector is defined as

(14) $$\Delta x={\left[\Delta h,\Delta \beta ,\Delta \lambda ,\Delta t_{0},\Delta t_{r,res},\Delta t_{c,res},\Delta d_{sub},\Delta d_{res}\right]}^{T},$$

where Δh denotes the radar ranging deviation; Δβ denotes the construction-angle deviation; Δλ denotes the inter-module geometric consistency deviation; $\Delta t_{0}$ denotes the common intersection-timing deviation; $\Delta t_{r,res}$ and $\Delta t_{c,res}$ denote the residual sampling deviation of the radar and camera relative to the common intersection instant, respectively; and the remaining two terms are defined above.

The effective input covariance matrix is defined as

(15) $$\sum_{x}=Cov\left(\Delta x\right).$$

The off-diagonal terms of $\sum_{x}$ represent physical correlations in the measurement chain. These correlations originate mainly from shared phase-triggering, shared geometric references, and coupled sensor-point definitions. For example, the angular encoder provides a common phase reference for both radar ranging and camera imaging; therefore, a phase deviation simultaneously shifts the effective sampling state of both modules. Similarly, uncertainty in the platform geometric reference or camera extrinsic parameters may affect both the vision-based construction angle and the inter-module angular registration.

The radar ranging deviation Δh and the sensor-point substitution deviation $\Delta d_{sub}$ are defined to represent different residual effects. Specifically, Δh describes the residual variation of the extracted range along the radar measurement direction after ranging calibration, whereas $\Delta d_{sub}$ describes the residual spatial mismatch between the effective sensor observation point and the geometrically defined blade-tip reference after deterministic point-location correction [24]. The range-direction component is therefore not assigned to $\Delta d_{sub}$. Accordingly, covariance between Δh and $\Delta d_{sub}$ is not introduced in the present covariance matrix due to the lack of independent experimental evidence. If a statistically supported coupling between the two residual quantities is identified in future measurements, the corresponding covariance term can be incorporated explicitly into Equation (15).

The principal effective deviations and their correlation mechanisms are summarized in Table 1.

### 3.2. Timing and Geometric Coupling

The phase-triggering chain establishes the temporal reference of the proposed measurement system. Ideally, the radar-measured blade-tip distance and the camera-derived blade-tip position represent the same blade-intersection state. In practice, phase determination, trigger judgment, module delay, and sampling-time variation shift the effective radar and camera measurements away from the theoretical intersection instant.

Let $t_{0}$ denote the theoretical blade-intersection instant and ω denote the rotor angular speed. For a phase-determination deviation Δθ, the corresponding common intersection-timing deviation is

(16) $$\Delta t_{0}=\frac{\Delta \theta }{\omega }.$$

The effective sampling instants of the radar and camera are expressed as

(17) $$t_{r}=t_{0}+\Delta t_{0}+\Delta t_{r,res},t_{c}=t_{0}+\Delta t_{0}+\Delta t_{c,res}.$$

Accordingly, the total timing deviations of the radar and camera relative to the theoretical intersection instant are

(18) $$\Delta t_{r}=\Delta t_{0}+\Delta t_{r,res},\Delta t_{c}=\Delta t_{0}+\Delta t_{c,res}.$$

Equations (17) and (18) show that the two measurement modules share the common timing component $\Delta t_{0}$. Therefore, the effective radar and camera timing deviations are intrinsically correlated even when the module-specific residual delays $\Delta t_{r,res}$ and $\Delta t_{c,res}$ are statistically independent. In addition, because both modules are controlled by the same FPGA-based triggering chain, a residual covariance between $\Delta t_{r,res}$ and $\Delta t_{c,res}$ may also remain.

Near the theoretical intersection instant t = $t_{0}$, the actual blade-tip distance acquired by the radar can be locally approximated as

(19) $$h\left(t_{r}\right)\approx h_{0}+\Delta h+{\dot{h}}_{0}\left(\Delta t_{0}+\Delta t_{r,res}\right),$$

where $h_{0}$ is the nominal distance at the theoretical intersection instant and ${\dot{h}}_{0}$ is the first-order temporal rate of the local blade-tip distance within the intersection window.

Similarly, the camera-derived construction angle is approximated as

(20) $$\beta \left(t_{c}\right)\approx \beta_{0}+\Delta \beta +{\dot{\beta }}_{0}\left(\Delta t_{0}+\Delta t_{c,res}\right),$$

where $\beta_{0}$ is the nominal construction angle at the theoretical intersection instant and ${\dot{\beta }}_{0}$ is the corresponding first-order temporal rate of the construction angle.

Under the present platform condition, ${\dot{h}}_{0}$ is estimated from a local first-order least-squares fit of the valid radar-distance samples within the phase-gated intersection window, using their effective timestamps relative to $t_{0}$. The fitting is performed on the range-versus-time sequence after FMCW range extraction. Therefore, no point-to-point numerical differentiation is used to estimate ${\dot{h}}_{0}$. At the nominal rotational speed of 420 r/min, the tested blade-intersection duration is approximately 7.74 ms; the same estimation procedure is applied to the shorter intersection window at 600 r/min, for which the duration is approximately 5.23 ms. Because the rotating blade and the co-rotating camera remain rigidly fixed relative to the rotor hub on the present platform, the nominal construction angle is constant within the intersection window. Accordingly, ${\dot{\beta }}_{0}$ is taken as zero, and no numerical differentiation of the camera-derived construction angle is performed in the present experiment. This assumption is specific to the current platform; for a deformable rotor system, ${\dot{\beta }}_{0}$ should instead be estimated from the local blade-tip motion.

Equations (19) and (20) explicitly distinguish residual sensor deviations from timing-induced dynamic deviations. The common phase error affects both h and β through $\Delta t_{0}$, whereas the module-specific timing errors act through $\Delta t_{r,res}$ and $\Delta t_{c,res}$, respectively.

The construction angle is obtained from the blade-tip position and platform geometric parameters according to the geometric relationship defined in Section 2.3. In a general form,

(21) $$\beta =g\left(P,\eta \right).$$

Equation (20) separates the observed angular deviation into a sensor residual and a modeled timing term. Because the effective camera timestamp and ${\dot{\beta }}_{0}$ are retained for each event, this timing term is calculated event by event and subtracted before covariance estimation. This prevents the same timing variation from being counted both in Δβ and in the explicit timing inputs. After the timing-induced component in Equation (20) is removed, the residual construction-angle deviation can be locally represented as

(22) $$\Delta \beta \approx \frac{\partial \beta }{\partial P}\Delta P+\frac{\partial \beta }{\partial \eta }\Delta \eta .$$

Similar to Δβ, the residual angular misalignment Δλ is also influenced by the installation and geometric-registration parameters. Therefore, camera extrinsic uncertainty, platform reference-point uncertainty, and radar installation-direction deviation will affect both Δβ and Δλ. Consequently, $Cov\left(\Delta \beta ,\Delta \lambda \right)\neq 0$ in the general case.

This covariance has a direct physical origin. These two effective deviations inherit a common perturbation from the geometric transformation and inter-module registration process. The corresponding covariance term is retained in the uncertainty propagation model as a result, and is evaluated from repeated geometric registration as described in Section 4.

### 3.3. First-Order Linearization

Substituting Equations (19) and (20) into the residual measurement model in Equation (13) gives

(23) $$\begin{array}{l} d=\left[h_{0}+\Delta h+{\dot{h}}_{0}\left(\Delta t_{0}+\Delta t_{r,res}\right)\right]\\ \times \cos\left[\beta_{0}+\Delta \beta +{\dot{\beta }}_{0}\left(\Delta t_{0}+\Delta t_{c,res}\right)+\Delta \lambda \right]\\ +\Delta d_{sub}+\Delta d_{res} \end{array}$$

Equation (23) describes the error-augmented reduced measurement model. It is distinguished from Equation (12) in the way that the latter defines the warning-parameter output, whereas Equation (23) is used to study only the propagation of the residual input uncertainty.

Linearizing Equation (23) around the nominal operating point ($h_{0},\beta_{0},\lambda_{0}=0,t_{0}$) and retaining first-order terms yields

(24) $$\begin{array}{l} \Delta d=J_{h}\Delta h+J_{\beta }\Delta \beta +J_{\lambda }\Delta \lambda \\ +J_{0}\Delta t_{0}+J_{r}\Delta t_{r,res}+J_{c}\Delta t_{c,res}\\ +\Delta d_{sub}+\Delta d_{res,} \end{array}$$

where $\Delta d=d-d_{0}$.

The sensitivity coefficients are given by

(25) $$J_{h}=\cos\beta_{0},$$

(26) $$J_{\beta }=-h_{0}\sin\beta_{0},$$

(27) $$J_{\lambda }=-h_{0}\sin\beta_{0},$$

(28) $$J_{0}={\dot{h}}_{0}\cos\beta_{0}-h_{0}{\dot{\beta }}_{0}\sin\beta_{0},$$

(29) $$J_{r}={\dot{h}}_{0}\cos\beta_{0},$$

(30) $$J_{c}=-h_{0}{\dot{\beta }}_{0}\sin\beta_{0}.$$

The construction-angle deviation Δβ and the angular-registration deviation Δλ represent different physical error sources, but both are signed in-plane angular perturbations and they enter Equation (23) additively through the same angular argument. Consequently, at the nominal condition $\lambda_{0}=0$, they have the same first-order sensitivity, $J_{\lambda }=J_{\beta }=-h_{0}\sin\beta_{0}$.

The additive residual components $J_{sub}$ and $J_{res}$ have unit sensitivity coefficients. The local sensitivity vector is therefore defined as $J=\left[J_{h},J_{\beta },J_{\lambda },J_{0},J_{r},\Delta J_{c},1,1\right]$. Using the effective input-deviation vector defined in Equation (14), Equation (24) can be written compactly as

(31) Δd=JΔx.

The first-order approximation in Equation (31) is local and requires small input perturbations and a stable fixed intersection pair. Its applicability under the present experimental conditions is examined by direct Monte Carlo propagation of the nonlinear model in Equation (23), as reported in Section 5.2 and Section 5.5. Boundary-point switching, which is absent from the present fixed-pair platform, is considered separately in Section 3.6.

### 3.4. Correlated Covariance Propagation

According to the first-order law of propagation of uncertainty, the combined variance of the warning parameter is obtained by propagating the covariance matrix of the effective input quantities through the local sensitivity vector:

(32) $$u_{c}^{2}\left(d\right)=J\sum_{x}J^{T}.$$

and the corresponding combined standard uncertainty is

(33) $$u_{c}\left(d\right)=\sqrt{J\sum_{x}J^{T}}.$$

Equation (32) is the core expression of the proposed correlated uncertainty propagation framework. It gives

(34) $$u_{c}^{2}\left(d\right)=\sum_{i=1}^{8}J_{i}^{2}u^{2}\left(x_{i}\right)+2\sum_{i<j}J_{i}J_{j}Cov\left(x_{i},x_{j}\right),$$

where $u\left(x_{i}\right)$ denotes the standard uncertainty associated with the i-th effective input quantity, and $Cov\left(x_{i},x_{j}\right)$ denotes the covariance between the i-th and j-th input quantities. The first term of Equation (34) represents the variance contributions associated with individual effective input quantities. The second term represents the contribution produced by physical correlation between input quantities. Depending on the signs of the sensitivity coefficients and covariance terms, a correlation may either increase or decrease the combined variance. Therefore, the magnitude of a correlation coefficient alone is insufficient to determine its effect on $u_{c}\left(d\right)$. The product $J_{i}J_{j}Cov\left(x_{i},x_{j}\right)$ must be considered.

The covariance matrix used in Equation (32) contains components evaluated by statistical analysis of synchronized repeated events and components evaluated by calibration certificates, repeated calibration, and other Type B information [25]. In addition, the assembled covariance matrix is symmetric and positive semidefinite before propagation.

For reporting the uncertainty of the warning parameter, the expanded uncertainty is defined as

(35) $$U\left(d\right)=k_{p}u_{c}\left(d\right),$$

where $k_{p}=2$ is used as an engineering coverage factor, which corresponds approximately to 95% coverage.

### 3.5. Covariance-Allocated Uncertainty Contributions

When the effective input quantities are correlated, ranking uncertainty sources solely according to the individual diagonal terms $J_{i}^{2}u^{2}\left(x_{i}\right)$ can be misleading because cross-covariance contributions are ignored. To identify dominant uncertainty paths while preserving the total propagated variance, the effective inputs are partitioned into physically meaningful uncertainty groups.

For the reduced measurement chain, the principal groups are defined as radar ranging, vision-based construction angle, inter-module geometric consistency, timing, sensor-point substitution, and model discrepancy. Let S denote one uncertainty group and −S denote all remaining groups. The sensitivity vector and covariance matrix are partitioned accordingly as $J=\left[J_{S},J_{-S}\right]$, and $\sum_{x}=\left[\begin{array}{cc} \sum_{SS} & \sum_{S,-S}\\ \sum_{-S,S} & \sum_{-S,-S} \end{array}\right]$. The signed covariance-allocated variance contribution of group S is defined as

(36) $$V_{S}=J_{S}\sum_{SS}J_{S}^{T}+J_{S}\sum_{S,-S}J_{-S}^{T},$$

The corresponding contribution ratio is

(37) $$C_{S}=\frac{V_{S}}{u_{c}^{2}\left(d\right)}\times 100\%.$$

With the definition in Equation (36), each cross-group covariance contribution is allocated equally between the two participating groups through the symmetry of the covariance matrix. Therefore, $\sum_{S}V_{S}=u_{c}^{2}\left(d\right)$, and $\sum_{S}C_{S}=100\%$.

### 3.6. Extension to the Complete Coaxial Twin-Rotor Model

The uncertainty propagation model in Section 3.1, Section 3.2, Section 3.3, Section 3.4 and Section 3.5 is derived for the reduced fixed-intersection-pair model used on the present test platform. In a complete coaxial twin-rotor system, the hazardous boundary points are selected from multiple upper and lower blade-tip candidates according to the minimum and maximum operations defined in Section 2.2. Consequently, the active measurement model may change when the selected boundary-point combination changes.

The warning parameter for a fixed boundary point combination $\chi =\left(I,J\right)$ can be written as $d_{\chi }=f_{\chi }\left(z\right)$, where z contains the measurements and system parameters required by the complete coaxial twin-rotor model.

When the axial coordinates of two or more candidate blade-tip points are close, small structural or measurement perturbations may change the result of the minimum or maximum operation. In this case, the active combination χ becomes a state-dependent discrete variable and a single fixed Jacobian no longer describes the complete output distribution. Let $p_{\chi }$ denote the probability that boundary-point combination is active. The mean warning parameter is then expressed as

(38) $$\mu_{d}=\sum_{\chi }p_{\chi }\mu_{\chi },$$

where $\mu_{\chi }$ is the conditional mean associated with combination χ.

To quantify the stability of the boundary-point selection, let $z_{a}$ denote the axial coordinate of the nominally selected boundary point and $z_{b}$ that of its nearest competing candidate. Their mean separation is defined as $\Delta z=\left|\mu_{z_{a}}-\mu_{z_{b}}\right|$, and the standard uncertainty of their difference is $u_{\Delta z}=\sqrt{u^{2}(z_{a})+u^{2}(z_{b})-2Cov(z_{a}-z_{b})}$. A normalized separation index is then defined as $\eta =\frac{\Delta z}{u\Delta z}$. Under a local Gaussian approximation for the candidate-coordinate difference, the probability that their nominal ordering is reversed is $P_{SW}=\Phi (-\eta )$, where Φ(·) denotes the cumulative distribution function of the standard normal distribution. For multiple competing candidates, the minimum value $\eta_{\min}$ over all relevant candidate pairs is used as the selection-stability indicator. In this study, we select $P_{SW}\geq 5\%$, equivalently $\eta_{\min}\leq 1.645$, as an operational criterion for non-negligible boundary-point switching. The 5% value is chosen as a pragmatic operational threshold, corresponding to the one-sided 95% confidence level under local Gaussian approximation. When the probability of ordering reversal reaches this level, the assumption of a fixed boundary-point ordering is no longer regarded as sufficiently reliable for uncertainty propagation, and the switching effect should be treated explicitly. Under this condition, a single fixed Jacobian is not used as the sole uncertainty-propagation model, and Monte Carlo propagation including the discrete boundary-point selection is required.

As an illustrative numerical example, consider two competing boundary-point coordinates with standard uncertainties $u(z_{a})=u(z_{b})=0.707$ mm and negligible covariance. According to the definition above, their coordinate-difference uncertainty is therefore $u_{\Delta_{z}}\approx 1.0$ mm. Let the nominal coordinate of the currently selected point be $\mu_{z_{a}}=10.0$ mm, while the nearest competing point takes $\mu_{z_{b}}=7.0,8.5,9.5$ mm in three representative cases. The resulting candidate separations are Δz=3.0,1.5,0.5 mm, corresponding to normalized separation indices of η=3.0,1.5,0.5 and switching probabilities of approximately 0.13%, 6.68%, and 30.85%, respectively. The first case represents a stable boundary-point selection, whereas the latter two exceed the adopted 5% criterion and therefore require propagation over the alternative boundary-point branches.

According to the law of total variance, the overall variance can be represented as

(39) $$u^{2}\left(d\right)=\sum_{\chi }p_{\chi }\left[u_{\chi }^{2}\left(d\right)+{\left(\mu_{\chi }-\mu_{d}\right)}^{2}\right].$$

To illustrate the effect of boundary-point switching on the warning-parameter distribution, consider two possible boundary-point combinations with conditional warning-parameter distributions having means of 10.0 and 7.0 mm, and the same within-branch standard uncertainty of 1.0 mm. Using the switching probabilities in the preceding example, Equation (39) gives overall means and standard uncertainties of 9.996 mm and 1.006 mm for $P_{SW}=0.13\%$, 9.800 mm and 1.249 mm for $P_{SW}=6.68\%$, and 9.075 mm and 1.709 mm for $P_{SW}=30.85\%$, respectively. Thus, once the switching probability becomes non-negligible, the output is no longer adequately represented by the uncertainty of a single local branch; instead, the warning parameter becomes a two-branch mixture whose mean shifts and whose dispersion increases because of between-branch variability. This example quantitatively demonstrates the effect of boundary-point switching on both the location and dispersion of the warning-parameter distribution.

In an actual coaxial twin-rotor system, the probabilities $p_{\chi }$ are generally determined jointly by rotor phase, blade deformation, aerodynamic loading, and measurement uncertainty. When the maximum switching probability satisfies the adopted criterion $P_{SW}\geq 5\%$, the active boundary-point combination is no longer treated as fixed, and branch-aware Monte Carlo propagation is used to account for both continuous measurement uncertainties and discrete boundary-point selection. Below this threshold, first-order covariance propagation may be applied to the stable active branch.

## 4. Experimental Uncertainty Evaluation

The uncertainty propagation model established in Section 3 requires a pointwise covariance matrix for the effective input deviations, and an independently constructed reference value for residual assessment. Accordingly, the experiments in this section focus on identifying the uncertainty information required by the propagation model. Event-resolved components such as Δh, Δβ, $\Delta t_{0}$, $\Delta t_{r,res}$, and $\Delta t_{c,res}$ are evaluated from synchronized blade-intersection data. Calibration-constrained components such as encoder phase calibration, radar static-ranging calibration, camera calibration, and inter-module geometric registration are evaluated from repeated calibration and Type B information.

### 4.1. Test Platform and Conditions

The experiments are conducted on the single-rotor blade-intersection test platform shown in Figure 4 and Figure 5. The platform provides repeatable short-duration blade-intersection events with a deterministic phase, an adjustable reference geometry, and synchronized acquisition windows. The phase, position, and distance measurements are obtained using an angular encoder, a tracking camera, and a millimeter-wave radar, respectively. Their outputs and effective timestamps are recorded by the multi-source information fusion unit. The detailed measurement architecture and fusion procedure are available in the preceding study [19].

The main uncertainty evaluation is performed at a rotational speed of 420 r/min. Ten reference points are established over the range of 99–990 mm, at intervals of 99 mm. Five repeated acquisition groups are recorded at each reference point, and each group contains 20 valid blade-intersection events. Thus, 100 valid events are available at each reference point, giving a total of 1000 synchronized event records for the main evaluation.

For each reference point, the reference warning parameter is constructed from the guide rail setting and the independently calibrated static platform geometry. Under the fixed-pair platform condition, the reference value is expressed as $d_{R,\iota }=h_{R,\iota }\cos\beta_{R,\iota }$, where $h_{R,\iota }$ is the reference blade-tip distance established at the ι−th measurement point and $\beta_{R,\iota }$ is the corresponding reference construction angle. The standard uncertainty of the reference value is propagated from the guide-rail positioning uncertainty, stationary-blade positioning uncertainty, and static geometric calibration uncertainty.

Supplementary measurements are conducted at 300 and 600 r/min at reference points of 99 mm, 495 mm, and 891 mm, representing the near-, middle-, and long-range conditions, respectively. These measurements are used to examine the dynamic robustness of the measurement chain. For the 600 r/min Monte Carlo validation, the event-derived variances and covariances are re-estimated separately from the corresponding synchronized data, while calibration-constrained Type B components, which are independent of rotational speed, retain their nominal evaluated values. These speed-specific statistics are used only for the 600 r/min validation, and are not incorporated into the covariance matrices established for the nominal 420 r/min condition.

### 4.2. Input Covariance Evaluation

At each reference point, the retained event-level data include the encoder phase, the effective radar and camera timestamps, the radar-measured blade-tip distance, the vision-based construction angle, and the fused warning parameter. The intermediate measurements and timestamps are retained to separate timing-induced variations before the effective input covariance matrix is estimated. These quantities have already been defined in Section 2 and Section 3.

Prior to covariance estimation, the timing-induced components identified by the model in Section 3.2 are removed from the measured distance and construction-angle variations. The remaining quantities correspond to the residual input deviations Δh and Δβ defined in Section 3.1.

At each reference point, the event-derived covariance terms are evaluated from five repeated acquisition groups, each containing 20 valid intersection events. To exclude slow offsets among repeated groups, each event-resolved effective input is first centered within its corresponding group:

(40) $${\tilde{\Delta x}}_{a,lm\nu }=\Delta x_{a,lm\nu }-\frac{1}{20}\sum_{\nu =1}^{20}\Delta x_{a,lm\nu }.$$

where l, m, and ν denote the reference point, repeated group, and event number, respectively.

For two event-resolved inputs $x_{a}$ and $x_{b}$, the pooled within-group covariance at reference point l is calculated as

(41) $$s_{ab,l}=\frac{1}{5(20-1)}\sum_{m=1}^{5}\sum_{\nu =1}^{20}{\tilde{\Delta x}}_{a,lm\nu }{\tilde{\Delta x}}_{b,lm\nu }.$$

The pointwise correlation coefficient is then obtained from the corresponding covariance-matrix entries:

(42) $$r_{ab,l}=\frac{{\left[\sum_{x,l}\right]}_{ab}}{\sqrt{{\left[\sum_{x,l}\right]}_{aa}{\left[\sum_{x,l}\right]}_{bb}}}.$$

For event-resolved variable pairs, the covariance entry in Equation (42) is obtained from Equation (41). For pairs involving calibration-constrained quantities, such as Δλ, the corresponding covariance is obtained from repeated geometric registration.

Calibration-constrained components are evaluated independently from repeated calibration, instrument calibration information, and installation measurements. The measured result will be corrected first when a systematic deviation is identified, and only the uncertainty associated with the correction is propagated. A known deterministic bias is therefore not treated as a zero-mean random uncertainty component. Shared calibration parameters may affect more than one effective input. For example, the same geometric reference contributes to both the construction-angle deviation and the residual angular-registration deviation. The corresponding covariance is retained in the calibration-derived covariance matrix.

To establish an experimental basis for the radar-related sensor-point substitution uncertainty, 10 independent near-field scattering-center scans are performed at each of the 10 reference distances used in the main uncertainty experiment. Each repeated scan independently reconstructs the dominant electromagnetic scattering-center coordinates in the blade-tip local $Y_{c}-Z_{s}$ (where $Y_{c}$ and $Z_{s}$ denote the chordwise and spanwise directions, respectively) plane relative to the geometrically defined blade-tip reference point. At a reference distance $d_{R}$, the corresponding sample covariance matrix is defined as

(43) $$\sum_{sc}(d_{R})=Cov\left(\left[\begin{array}{l} Y_{c}\\ Z_{s} \end{array}\right]\right),$$

The mean reconstructed coordinates at each reference distance are used as deterministic point-location corrections. The uncertainty associated with scattering-center substitution is evaluated from the repeat-scan covariance after these mean corrections are applied. As the residual range-direction uncertainty is already included in Δh, a conservative scalar measure of the in-plane scattering-center repeatability is defined as

(44) $$u_{sc}(d_{R})=\sqrt{\lambda_{\max}\left[\sum_{sc}\left(d_{R}\right)\right]},$$

To provide an independent experimental basis for the residual model-discrepancy component, a geometric model-closure evaluation is performed separately from the dynamic residual assessment. At each reference distance, the blade-intersection geometry is reconstructed using an independent static geometric reference, and the warning parameter obtained from this representation is compared with that calculated using the reduced relation d=hcosβ. Ten independent closure evaluations are performed at each reference distance. For the k−th evaluation at reference point $d_{R,l}$, the closure residual and the associated standard uncertainty are defined as

(45) $$\left\{\begin{array}{l} r_{cl,l,k}=d_{geo,l,k}-d_{red,l,k}\\ u(\Delta d_{res,l})=\sqrt{s_{cl,l}^{2}+u_{cl,ref,l}^{2}} \end{array}\right.,$$

where $s_{cl,l}$ is the sample standard deviation of the repeated closure residuals and $u_{cl,ref,l}$ is the standard uncertainty associated with the independent geometric reference used for the closure evaluation.

As shown in Table 2, over the reference-distance range of 99–990 mm, $u_{sc}$ generally increases from 0.143 to 0.342 mm, consistent with the increasing sensitivity of the effective scattering-center location as the radar illumination footprint expands. A distinct local behavior is observed at 891 mm, where $u_{sc}$ increases to 0.487 mm. The reconstructed mean scattering-center coordinate at this point also shifts to (−6.819, 51.169) mm, compared with (−6.610, 50.891) mm at 792 mm and (−6.661, 50.911) mm at 990 mm. The simultaneous increase in repeatability dispersion and local mean-position migration indicates a state-dependent change in the dominant scattering response.

$u_{sc}$ is evaluated from 10 independent repeated near-field scattering-center reconstructions at each reference distance using the maximum directional standard deviation derived from the two-dimensional covariance matrix. The geometric point-substitution uncertainty $u_{geo-sub}$ is evaluated from the common target-to-tip geometric calibration, and is therefore treated as distance independent over the tested range. The sensor-point substitution uncertainty is calculated as $u(\Delta d_{sub})=\sqrt{u_{sc}^{2}+u_{geo-sub}^{2}}$.

The independently evaluated sensor-point substitution uncertainty shows a generally increasing trend with measurement distance, but it is not strictly monotonic. The combined standard uncertainty $u(\Delta d_{sub})$ increases from 0.230 mm at 99 mm to 0.386 mm at 990 mm under the nominal geometric point-calibration uncertainty. A pronounced local increase is observed at 891 mm, where $u(\Delta d_{sub})$ reaches 0.519 mm, aligned with the increase found in the repeat-scan scattering-center component. This result indicates that the sensor-point substitution contains a range-dependent component together with localized state-dependent scattering behavior.

The independently evaluated residual model-discrepancy uncertainty increases from 0.306 mm at 99 mm to 0.459 mm at 990 mm. The gradual increase in $u(\Delta d_{res})$ is mainly associated with the increasing dispersion of the geometric closure residual and the distance-dependent projection of the independent reference-direction uncertainty. No distinct anomaly is observed at 891 mm.

### 4.3. Residual Compatibility

For the ν−th valid event at reference point ι, the dynamic measurement residual is defined as

(46) $$e_{\iota ,\upsilon }=d_{\iota ,\upsilon }-d_{R,\iota },$$

This event-level definition ensures that the residual and the propagated uncertainty refer to the same measurement object. The pointwise mean residual is used separately as a descriptive indicator of bias tendency.

Although the reference value is constructed through a separate static route, it may share geometric calibration quantities with the dynamic measurement result. The covariance associated with these shared inputs is therefore retained. The variance of the event-level residual is

(47) $$u^{2}\left(e_{\iota ,\upsilon }\right)=u_{c}^{2}\left(d_{\iota }\right)+u^{2}\left(d_{R,\iota }\right)-2Cov\left(d_{\iota ,\upsilon },d_{R,\iota }\right),$$

where $u_{c}\left(d_{\iota }\right)$ is obtained by propagating $\sum_{x,\iota }$ through the model established in Section 3, Equation (32).

The residual is regarded as compatible with the propagated uncertainty when $\left|e_{\iota ,\upsilon }\right|\leq k_{p}u\left(e_{\iota ,\upsilon }\right)$, $k_{p}$ = 2.

The pointwise mean residual, repeatability standard deviation, and coefficient of variation are calculated from the five repeated acquisition groups. The mean residual is not compared directly with $U\left(d\right)$ as a coverage statement. This is because such a comparison would require a separate uncertainty evaluation for the mean output, distinguishing event-level random components from calibration and reference quantities shared by all repeated measurements.

## 5. Results and Discussion

Based on the measurement and uncertainty-evaluation procedures presented in Section 3 and Section 4, experiments are conducted on the single-rotor blade-intersection platform. This section reports the synchronized event quantities, pointwise covariance estimates, propagated uncertainty, residual compatibility, and supplementary multi-speed results. The effective-input covariance matrix is evaluated separately at each reference point according to Section 4, and the resulting pointwise matrices are propagated through the reduced measurement model. The results are presented in the order of input behavior, propagated output uncertainty, residual compatibility, uncertainty-source contribution, and multi-speed robustness.

### 5.1. Input Uncertainties and Correlations

**Figure 6:**
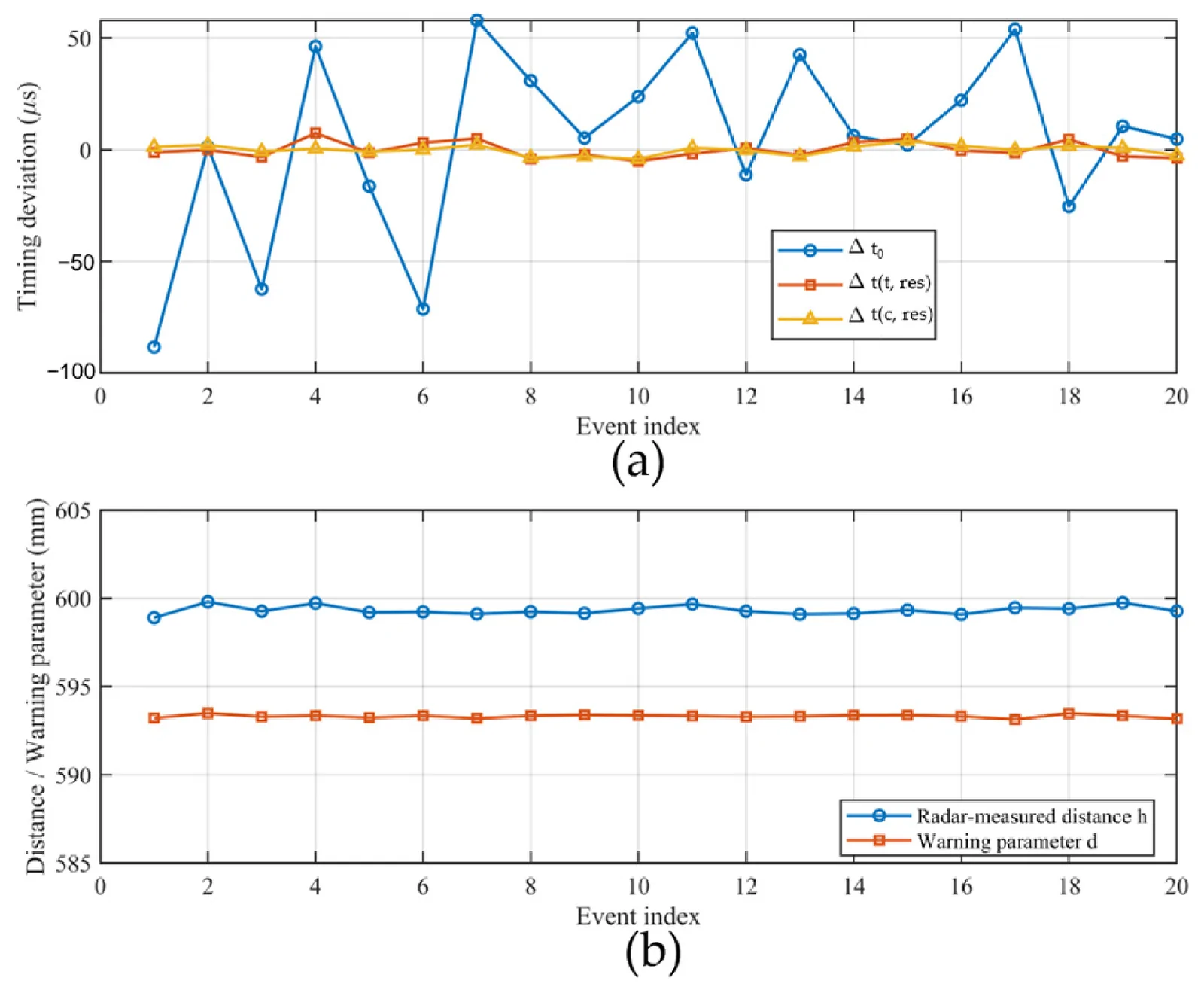
(**a**) Intersection-timing deviation and residual radar and camera sampling-time deviations. (**b**) Radar-measured blade-tip distance and calculated final warning parameter obtained from 20 synchronized blade-intersection sequences, at $d_{R}=594\;\mathrm{mm}$.

For each event, common intersection-timing deviation $\Delta t_{0}$ is calculated from the encoder phase deviation using Equation (16). The residual radar and camera sampling-time deviations are calculated from the differences between their effective acquisition timestamps and the trigger timestamp after subtracting the independently calibrated deviations, as defined in Equations (17) and (18). Figure 6a plots these three event-level quantities for one group of 20 individual events at $d_{R}=594\;\mathrm{mm}$. The fluctuation of the common phase-induced timing deviation is larger than those of the two module-specific residual timing terms. This is because the principal timing variation under the nominal operating condition originates from both the phase-determination process and shared phase-triggering path, whereas the residual timing delays of the radar and camera relative to the shared triggering reference remain comparatively small. Figure 6b presents the radar-measured blade-tip distance h and the warning parameter d for 20 individual blade-intersection events from one representative acquisition group at $d_{R}=594\;\mathrm{mm}$. Over the selected sequence, neither quantity exhibits a monotonic drift or an isolated large deviation. The warning parameter d is consistently 6 mm less than the radar-measured distance h because the latter is projected through the construction angle according to Equation (8). The approximately constant separation between the two curves is consistent with a relatively stable construction angle during the selected events. These results demonstrate stable event-level outputs under repeated blade-intersection conditions. This behavior is consistent with the sensitivity structure established in Section 3: under the small-construction-angle condition, the distance sensitivity $J_{h}$ is close to unity, and radar ranging variation is therefore transferred almost proportionally to the warning parameter.

After the timing-induced components are removed, the pointwise correlation coefficients are calculated using Equations (40)–(42). Ten coefficients are obtained independently for each variable pair at the ten reference points. Their arithmetic mean and minimum-to-maximum range are reported in Table 3. The correlation between Δh and Δβ remains weak, with a range-averaged coefficient of 0.004. The correlations of Δh and Δβ with the residual angular-registration deviation Δλ are more pronounced, with mean coefficients of 0.174 and 0.313, respectively. The largest estimated correlation is observed between the residual radar and camera timing deviations, with a mean coefficient of 0.442. This timing correlation is consistent with the shared FPGA triggering path.

The directly estimated correlations confirm that the effective inputs cannot all be treated as statistically independent. It should be noted that the magnitude of a correlation coefficient alone does not determine its contribution to the output variance. Its actual influence depends on the signs and magnitudes of the associated sensitivity coefficients as well. The propagated effects of these correlations are evaluated in Section 5.2 and Section 5.4.

### 5.2. Propagated Uncertainty

Table 4 presents the effective-input uncertainty budget at representative near-, middle-, and long-range reference points. Radar ranging is evaluated using the residual event variation and calibration information after timing-induced effects are separated. The construction-angle and angular-registration components are obtained from event data and repeated geometric registration, whereas the timing components are evaluated from the common phase deviation and the residual module timestamps. The sensor-point substitution uncertainty is evaluated independently from repeated near-field scattering-center reconstruction together with the calibrated target-to-tip geometry, whereas the residual model-discrepancy uncertainty is obtained from the independent geometric model-closure evaluation described in Section 4.2.

Table 4 shows that the standard uncertainty of the residual radar-ranging deviation increases from 0.525 mm at 99 mm to 0.777 mm at 990 mm. The sensor-point substitution uncertainty is constrained by repeated scattering-center reconstruction together with the common geometric point-calibration uncertainty. Its representative values are 0.230, 0.282, and 0.386 mm at 99, 495, and 990 mm, respectively. By contrast, the angular-registration uncertainty remains nearly constant at approximately 0.92–0.93 mrad. The increase in u(Δh) from 0.525 mm at 99 mm to 0.777 mm at 990 mm is consistent with the effect of the finite radar beamwidth on an extended blade-tip target. As the measurement distance increases, the illuminated footprint becomes larger, so variations in the composite echo profile and dominant range response can increase the range-axis variability associated with beat-frequency estimation and peak extraction. This range-axis effect is retained in Δh. By contrast, the spatial migration of the dominant electromagnetic scattering center relative to the geometrically defined blade-tip reference point is evaluated separately through $\Delta d_{sub}$ using the near-field reconstruction described in Section 4.2, thereby avoiding double counting of the two effects. These results indicate that the propagated uncertainty is governed primarily by radar ranging, sensor-point substitution, and residual model discrepancy, while the timing and angular-registration terms provide comparatively small adjustments.

**Figure 7:**
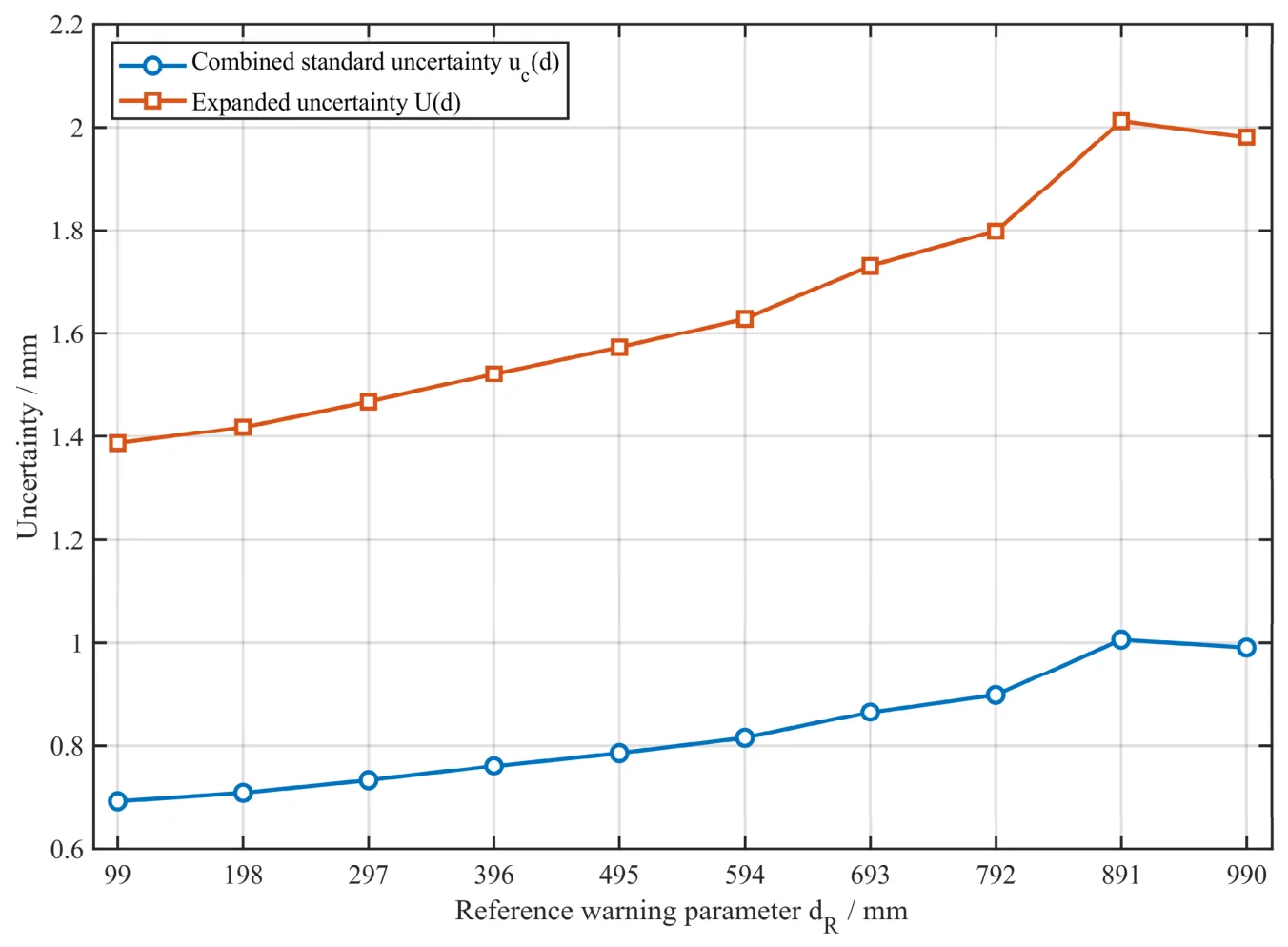
Pointwise combined standard uncertainty (blue circle) and expanded uncertainty (red square).

The input uncertainties in Table 4 and the correlations reported in Table 3 jointly determine the pointwise uncertainty through the covariance-propagation model in Equation (34). After incorporating the independently reassessed sensor-point substitution and residual model-closure components, the combined standard uncertainty ranges from 0.677 to 0.996 mm over the tested reference distances, while the corresponding expanded uncertainty ranges from 1.354 to 1.993 mm for $k_{p}=2$, as shown in Figure 7. The uncertainty generally increases with measurement distance. A localized maximum occurs at 891 mm, where $u_{c}(d)=0.996$ mm and U(d)=1.993 mm, followed by slightly lower values of 0.982 and 1.963 mm, respectively, at 990 mm. This local increase originates primarily from the independently identified increase in scattering-center repeatability at 891 mm.

To verify the validity of the first-order covariance propagation, Monte Carlo simulations are performed using the nonlinear measurement model, and the resulting uncertainties are compared with the first-order results at representative measurement distances. The first-order propagation is further validated by Monte Carlo propagation of the nonlinear model in Equation (23). After deterministic corrections, the event-derived and repeated-calibration residual quantities are sampled from a zero-mean multivariate normal distribution using the pointwise covariance matrix $\sum_{x}$, thereby retaining the measured correlations. For Type B quantities, calibration-certificate uncertainties are represented by normal distributions after conversion to standard uncertainties, whereas quantities specified only by symmetric bounded tolerances are represented by rectangular distributions over their stated limits. For each reference condition, $N_{MC}=10^{5}$ correlated input vectors are generated and propagated directly through Equation (23). The standard deviation of the resulting warning-parameter samples is denoted by $u_{MC}$.

At 420 r/min, the representative near-, middle-, and long-range conditions of 99, 495, and 891 mm give first-order combined standard uncertainties of 0.677, 0.769, and 0.996 mm, respectively. The corresponding Monte Carlo results are 0.678, 0.770, and 0.999 mm, giving relative differences of 0.15%, 0.13%, and 0.30%, respectively. The close agreement indicates that higher-order terms have a negligible influence under the nominal fixed-pair condition, and that the first-order covariance propagation provides an adequate approximation over the investigated uncertainty range.

### 5.3. Repeatability and Residual Compatibility

Table 5 summarizes the pointwise mean output, mean residual, repeatability standard deviation, and coefficient of variation. Over the tested range, the mean residual varies from −1.468 to 1.868 mm. The largest positive mean residual occurs at 891 mm, whereas the largest negative mean residual occurs at 990 mm. The maximum repeatability standard deviation is 0.615 mm, and the maximum coefficient of variation is 0.181%.

**Figure 8:**
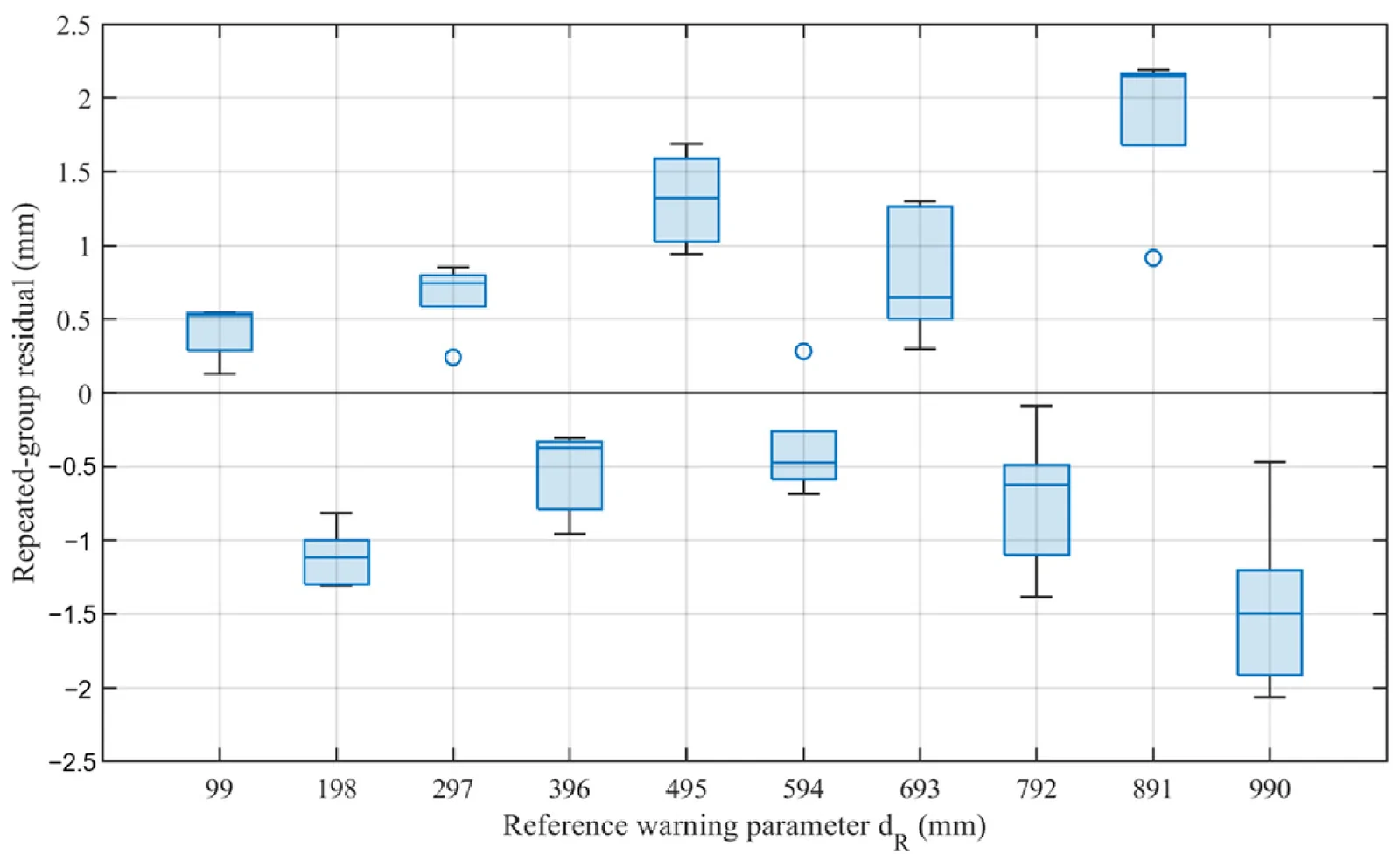
Distribution of repeated-measurement residuals at different reference points. The blue circles denote observations outside the whiskers.

Figure 8 presents the distribution of the repeated-group residuals at each reference point. Both positive and negative residuals occur over the measurement range, and no monotonic unidirectional trend is observed with increasing reference distance. The 891 mm results are predominantly positive, whereas the 990 mm results are predominantly negative and exhibit the largest repeatability dispersion. The alternating residual signs indicate that the observed deviations are consistent with point-dependent effects, such as local changes in radar scattering behavior, geometric registration, and reference construction. The larger dispersion at 990 mm is also consistent with the increase in radar-ranging uncertainty and angular sensitivity at the long-range end of the tested interval. Overall, these results demonstrate stable millimeter-level repeatability under the nominal operating condition.

**Figure 9:**
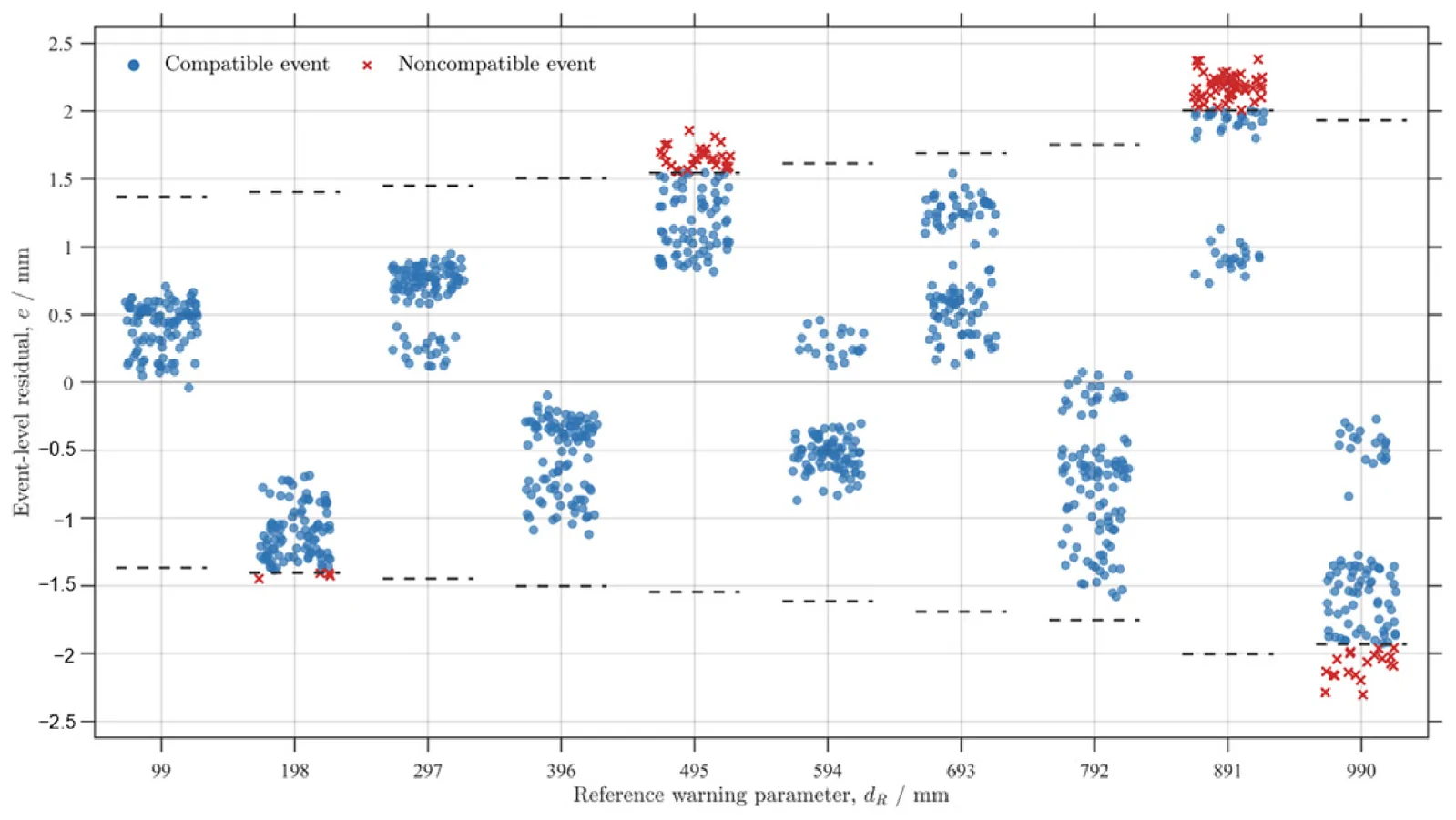
Event-level residuals and pointwise expanded residual-uncertainty limits at the 10 reference points. The dashed lines indicate the upper and lower expanded residual-uncertainty limits.

Figure 9 compares the event-level residuals with the corresponding expanded residual-uncertainty limits at the 10 reference points. After independently reassessing both the sensor-point substitution and residual model-closure components, 868 of the 1000 valid blade-intersection events satisfy the compatibility criterion, corresponding to an overall compatibility rate of 86.8%. Complete compatibility is obtained at 99, 297, 396, 594, 693, and 792 mm, while the compatibility rate at 198 mm is 91%. The rates at 495, 891, and 990 mm are 65%, 32%, and 80%, respectively.

The remaining localized non-compatibility exhibits different characteristics at the three most affected reference distances. At 495 mm, the mean residual is +1.310 mm, whereas the repeatability standard deviation is only 0.218 mm. The relatively small dispersion together with the predominantly positive residuals indicates a persistent point-dependent offset. Because the independently evaluated scattering-center repeatability at this distance follows the general range-dependent trend, possible contributors include residual reference-construction error, geometric-registration error, or acquisition-group offset.

At 891 mm, the mean residual increases to +1.868 mm. To investigate this localized anomaly, the experimental records and test conditions at this reference point were re-examined. No intentional change was introduced in the sensor configuration, platform geometry, rotational speed, or acquisition procedure, and no corresponding abnormal variation was observed in the phase, timing, or geometric quantities. We believe the abnormal behavior comes from the radar-related measurement branch. In the independent near-field reconstruction tests, the scattering-center repeatability increases locally to $u_{sc}=0.487$ mm, and the mean reconstructed scattering-center position also migrates relative to those at the neighboring reference points. The simultaneous occurrence of the radar-related residual anomaly, increased scattering-center dispersion, and local mean-position migration suggests that the reduced compatibility at 891 mm is primarily associated with a localized change in the electromagnetic response of the blade-tip target under this specific range and measurement condition. This behavior is consistent with instability of the dominant electromagnetic scattering center, which can alter both the effective radar observation point and the extracted range response.

At 990 mm, the mean residual changes to −1.468 mm and the repeatability standard deviation increases to 0.615 mm. The reduced compatibility at this point therefore reflects both a persistent negative offset and increased event-to-event dispersion. The increased dispersion is consistent with the larger long-range radar-ranging uncertainty and scattering-center variability, whereas the persistent negative offset may additionally involve residual reference-geometry or guide-rail alignment effects.

No deterministic polynomial correction is fitted to the pointwise residuals used for compatibility assessment. The residual means over the 10 reference distances do not exhibit a monotonic or independently demonstrated smooth dependence on $d_{R}$: instead, the affected reference points show different statistical and physical characteristics, as discussed above. Moreover, fitting a correction to the same event data subsequently used for residual-compatibility assessment would use the validation dataset for both calibration and validation, and would therefore compromise the independence of the assessment.

In an actual coaxial twin-rotor system, blade deformation, aerodynamic interaction, and changing sensing geometry introduce additional system state-dependence. A positive residual means that the measured warning parameter overestimates the safety clearance and may therefore delay a collision warning, whereas a negative residual produces a conservative clearance estimate and may increase the false-alarm rate. A more conservative implementation would compare the lower uncertainty bound, such as $d-U\left(d\right)$, with the warning threshold, and would update the uncertainty model according to geometric state.

Consider a notional warning threshold of $d_{th}=100$ mm as an example, and a measured warning parameter of d=101.5 mm with U(d)=2.0 mm. A decision that is based only on the nominal measured value would not issue a warning because $d>d_{th}$. On the contrary, the lower uncertainty bound in this example is d−U(d)=99.5 mm, and this is below the threshold and would therefore trigger a conservative warning. This example illustrates how incorporating measurement uncertainty can reduce the risk of a missed warning when the measured clearance is close to the decision threshold, although the corresponding trade-off is an increased possibility of conservative or false warnings.

### 5.4. Uncertainty Contributions

The signed covariance-allocated contributions in Table 6 are calculated using the group-allocation method defined in Section 3.5. The contribution assigned to each uncertainty group contains its within-group variance together with its allocated cross-group covariance terms. Consequently, individual group contributions may be negative when the corresponding covariance produces a net reduction in the propagated output variance. Following the independent assessment of both the sensor-point substitution and residual model-closure components, these two uncertainty groups are reported separately in the contribution analysis.

The radar-ranging group is the largest uncertainty contributor, with a mean signed contribution of 56.028%. The residual model-discrepancy and sensor-point substitution groups contribute 23.121% and 21.159%, respectively. The vision-based construction-angle, geometric-registration, and timing groups have mean signed contributions of −0.020%, −0.290%, and 0.001%, respectively. The small negative angular contributions result from covariance-induced cancellation.

A sensitivity analysis is further performed by simultaneously scaling the independently evaluated $u(\Delta d_{sub})$ and $u(\Delta d_{res})$ to 80%, 100%, and 120% of their nominal values while retaining the other uncertainty components unchanged. As shown in Table 7, the resulting combined standard uncertainty ranges are 0.637–0.913, 0.677–0.996, and 0.723–1.094 mm, respectively. The maximum change relative to the nominal result remains within 9.8%. When the two components are varied separately by ±20%, $u_{c}(d)$ is recalculated at all 10 reference points using Equation (32), with all other inputs retained at their nominal values. Taking the maximum absolute relative change over the tested reference points and the two scaling directions gives 5.9% for sensor-point substitution and 5.0% for residual model closure. The dominant uncertainty-source ranking remains unchanged, with radar ranging remaining the largest contributor. These results indicate that the principal uncertainty-budget conclusion is not sensitive to moderate variations in the two independently evaluated components.

To further evaluate the proposed correlated uncertainty model, we ran a conventional independent-input analysis at the 99 mm reference point by neglecting input correlations. In the conventional independent-input analysis, all off-diagonal elements of the pointwise covariance matrix in Equation (32) are set to zero, while the same standard uncertainties and sensitivity coefficients are retained. The corresponding combined standard uncertainty is therefore calculated as $u_{c,ind}=\sqrt{\sum_{i}J_{i}^{2}u_{i}^{2}}$. At 99 mm, this treatment gives $u_{c,ind}=0.646$ mm comparing to $u_{c,corr}=0.677$ mm when considering the correlations among different measurement sources. For this representative case, retaining the measured covariance terms changes the combined standard uncertainty from 0.646 mm to 0.677 mm, corresponding to a 4.80% increase relative to the independent-input result. Under the independent-input assumption, the contribution of each source is determined only from its diagonal variance term $J_{i}^{2}u_{i}^{2}/u_{c,ind}^{2}$. At this reference point, radar ranging, residual model closure, and sensor-point substitution account for approximately 64.8%, 22.4%, and 12.7% of the diagonal propagated variance, respectively.

By comparing the signed contributions reported in Table 6 and these diagonal fractions, we can see that the correlated uncertainty model allows covariance to either increase or reduce the contribution assigned to an uncertainty path, as indicated by the small negative contributions of some angular components. The advantage of the correlated model is not that it necessarily yields a larger or smaller uncertainty, but rather that it preserves the experimentally supported coupling among the phase, geometric, timing, and ranging inputs. As a result, it provides a more physically consistent estimate of both the combined uncertainty and the relative contributions of the individual uncertainty paths.

### 5.5. Multi-Speed Evaluation

**Figure 10:**
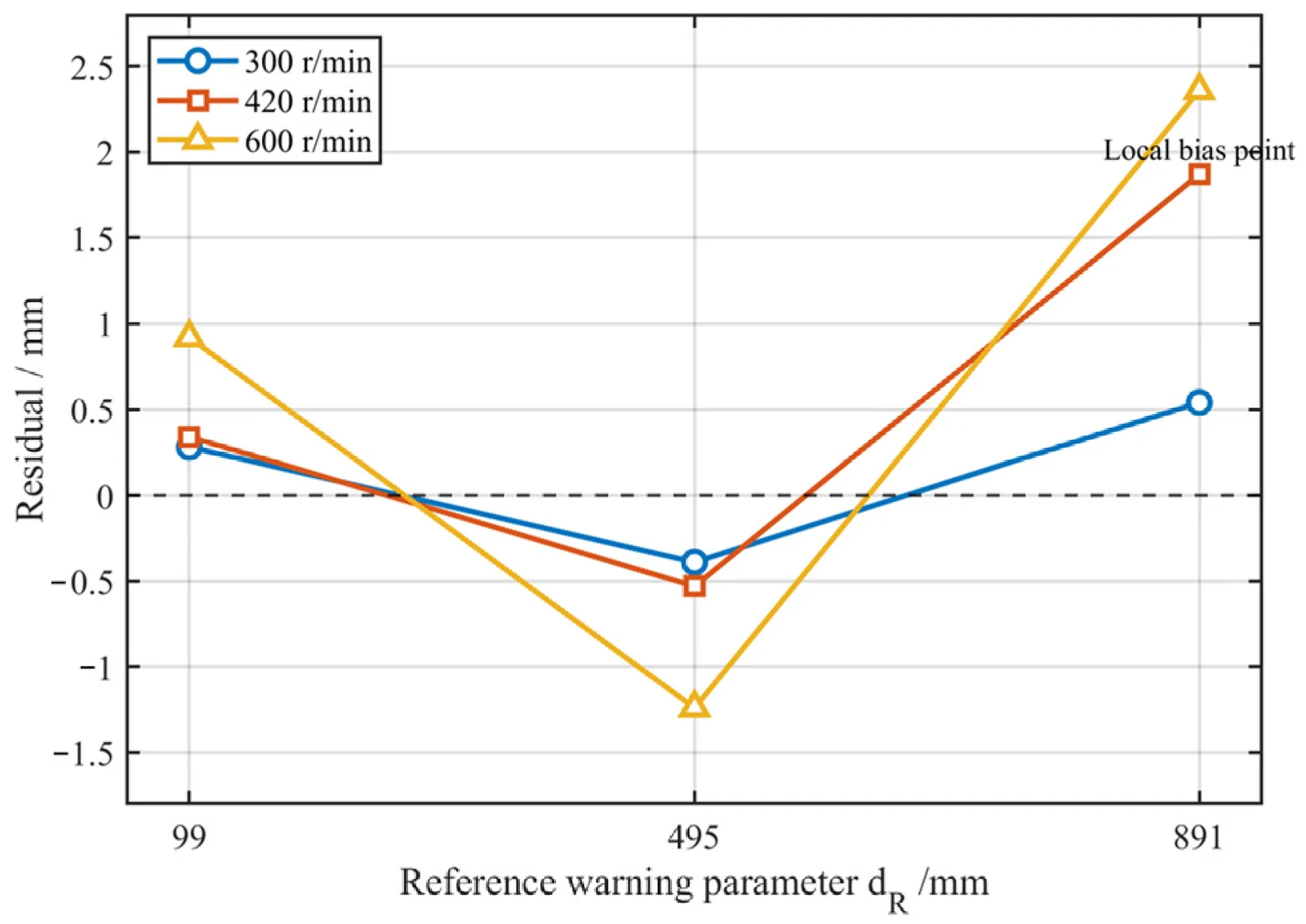
Multi-speed validation of dynamic measurement residuals at representative reference distances.

Supplementary measurements at 300 and 600 r/min are compared with the nominal 420 r/min results at the representative reference distances of 99, 495, and 891 mm. As shown in Figure 10, the residual ranges are approximately −0.89 to 0.67 mm at 300 r/min, −0.54 to 1.87 mm at 420 r/min, and −2.54 to 2.36 mm at 600 r/min. The residual magnitude therefore increases at some points under the highest tested speed, but remains within the millimeter range over the investigated conditions. Under the single-rotor blade-intersection platform conditions, these residual levels are acceptable for demonstrating that the measurement chain remains operational and retains millimeter-level robustness over the tested speed range. In real operation, acceptability must be assessed against the available safety margin and warning threshold, while also accounting for additional effects such as blade deformation, aerodynamic interaction, and time-varying sensing geometry. These mechanisms are absent from the present rigid fixed-pair covariance population and may introduce additional variances as well as cross-covariances among the phase, geometric, timing, and ranging inputs. Therefore, the expanded uncertainty U(d) reported in this study should be interpreted as conditional on the present single-rotor blade-intersection platform configuration, and may underestimate the system-level uncertainty of a complete coaxial twin-rotor system.

Compared with the 420 r/min main experiment, the 300 r/min condition has a longer interval between intersection events and a larger triggering time margin, so the residual magnitude is smaller. At 600 r/min, the intersection duration is shortened from 7.74 ms to 5.23 ms. As a result, the radar effective sampling window and vision-triggering timing become more restrictive for system stability. Consequently, the absolute residuals and repeatability standard deviations increase at some measurement points. Nevertheless, the current fusion measurement chain maintains acceptable dynamic robustness over the tested rotational-speed range.

To examine whether the shorter intersection window affects the validity of the linearized uncertainty propagation, the same Monte Carlo procedure is also applied to the highest tested speed of 600 r/min using the corresponding speed-specific input statistics. At 99, 495, and 891 mm, the first-order combined standard uncertainties are 0.731, 0.862, and 1.118 mm, respectively, while the corresponding Monte Carlo results are 0.733, 0.866, and 1.126 mm. The relative differences are 0.27%, 0.46%, and 0.72%, respectively. Although the discrepancy increases with measurement distance and rotational speed, it remains below 1% for all three representative conditions. The first-order propagation is therefore retained for the present fixed-pair measurement model over the tested speed range.

For a direct comparison with representative blade-clearance measurement techniques, Table 8 summarizes the reported dynamic performance of optical and ultrasonic approaches together with the present fusion measurement chain. Because the published studies use different measurands and performance metrics, the original reported error or uncertainty measure is retained rather than converted to a common uncertainty definition.

The comparison shows that existing optical and ultrasonic methods provide valuable direct measurements of blade-tip motion or inter-blade distance, but their reported performance is generally expressed at the individual sensing-output level. The present method does not aim to outperform all individual sensors in a single metric; instead, it provides synchronized phase, position, and distance fusion, and propagates their coupled uncertainties to the final blade collision warning parameter. Its present combined standard uncertainty remains below 1.0 mm over the tested 99–990 mm range.

## 6. Conclusions

This study establishes a correlated uncertainty-propagation framework for the phase–position–distance fusion measurement chain used to determine the blade collision warning parameter of coaxial twin-rotor helicopters. The framework propagates the effective ranging, geometric, timing, sensor-point substitution, and residual model-closure deviations to the final measurand, while explicitly retaining physically supported covariance terms. Sensor-point substitution and residual model closure are evaluated through independent near-field scattering-center reconstruction and geometric model-closure tests, respectively. A signed covariance-allocation method is further used to identify the net contribution of each uncertainty path without double counting cross-group covariance.

Under the nominal 420 r/min condition, 1000 synchronized intersection events are evaluated over the 99–990 mm range. The combined standard uncertainty is 0.677–0.996 mm and the corresponding expanded uncertainty is 1.354–1.993 mm for $k_{p}=2$, while 868 of the 1000 events satisfy the expanded residual-uncertainty criterion, giving an overall compatibility rate of 86.8%. Nonlinear Monte Carlo propagation agrees with the first-order results within 0.30% at 420 r/min and within 0.72% at the highest tested speed of 600 r/min. Radar ranging remains the dominant uncertainty path, with a mean signed contribution of 56.028%, followed by residual model closure (23.121%) and sensor-point substitution (21.159%). Simultaneously varying the latter two components by ±20% changes $u_{c}(d)$ by no more than 9.8%, and does not alter the dominant-source ranking.

These results provide three practical implications for system design. First, improvement of the warning-parameter uncertainty should prioritize radar range extraction and control of the effective electromagnetic observation point. Second, collision-warning decisions should use a conservative clearance such as d−U(d). Third, for a complete coaxial twin-rotor system, the fixed-branch first-order model should be replaced by branch-aware Monte Carlo propagation when the estimated boundary-point switching probability satisfies $P_{SW}\geq 5\%$. The uncertainty intervals reported here are conditional on the present platform and should not be interpreted as aircraft-level coverage intervals, because counter-rotation, aeroelastic deformation, aerodynamic interaction, and state-dependent sensing geometry introduce additional variance and covariance components that require validation on a complete coaxial rotor system.

## Figures and Tables

**Table 1:** Effective input deviations and principal correlation mechanisms in the reduced measurement chain.

Effective Deviation	Principal Physical Origin	Main Correlation Mechanism
Δh	Radar beat-frequency estimation, peak extraction, calibration residual, dynamic ranging fluctuation	Vibration, radar alignment, scattering behavior
Δβ	Camera calibration, target recognition, installation residual	Shared extrinsic and platform geometry
Δλ	Residual angular registration between radar ranging and construction directions	Common geometric reference and installation parameters
$\Delta t_{0}$	Encoder phase deviation and phase-trigger judgment	Common phase reference for radar and camera
$\Delta t_{r},\Delta t_{c}$	Module-level residual sampling delay and timestamp variation	Shared FPGA triggering chain
$\Delta d_{sub}$	Radar scattering-point and visual target-point substitution	Common point-reference geometry, where supported by calibration data
$\Delta d_{res}$	Residual closure difference between the independently calibrated geometric representation and the reduced model	Treated independently unless a shared calibration parameter is explicitly identified

**Table 2:** Independently evaluated components of sensor-point substitution and residual model discrepancy.

$d_{R}$/mm	${\bar{Y}}_{c}$/mm	${\bar{Z}}_{s}$/mm	$u_{sc}$/mm	$u_{geo-sub}$/mm	$u(\Delta d_{sub})$/mm	$s_{cl}$/mm	$u_{cl,ref}$/mm	$u(\Delta d_{res})$/mm
99.000	−6.341	50.619	0.143	0.180	0.230	0.300	0.060	0.306
198.000	−6.370	50.648	0.151	0.180	0.235	0.312	0.065	0.319
297.000	−6.399	50.688	0.172	0.180	0.249	0.326	0.073	0.334
396.000	−6.449	50.739	0.191	0.180	0.262	0.340	0.083	0.350
495.000	−6.490	50.799	0.217	0.180	0.282	0.355	0.094	0.367
594.000	−6.531	50.830	0.241	0.180	0.301	0.369	0.106	0.384
693.000	−6.570	50.860	0.272	0.180	0.326	0.384	0.119	0.402
792.000	−6.610	50.891	0.302	0.180	0.352	0.399	0.132	0.420
891.000	−6.819	51.169	0.487	0.180	0.519	0.414	0.146	0.439
990.000	−6.661	50.911	0.342	0.180	0.386	0.430	0.160	0.459

**Table 3:** Pointwise correlation coefficients of the effective input deviations.

Component Pair	Range	Mean
Δh,Δβ	[−0.083, 0.061]	0.004
Δh,Δλ	[0.131, 0.215]	0.174
Δβ,Δλ	[0.258, 0.351]	0.313
$\Delta t_{r,res},\Delta t_{c,res}$	[0.389, 0.508]	0.442

**Table 4:** Effective-input uncertainty budget at representative reference points.

Effective Input	Evaluation Route	$u\left(x\right)$, 99 mm	$u\left(x\right)$, 495 mm	$u\left(x\right)$, 990 mm
Δh/mm	Synchronized events + radar calibration	0.525	0.597	0.777
Δβ/mrad	Events + camera calibration	0.255	0.297	0.333
Δλ/mrad	Repeated geometric registration	0.930	0.928	0.922
$\Delta t_{0}$/μs	Phase-trigger-chain evaluation	54.0	42.1	49.4
$\Delta t_{r,res}$/μs	Radar timestamp statistics	2.38	2.83	2.46
$\Delta t_{c,res}$/μs	Camera timestamp statistics	2.48	2.22	2.39
$\Delta d_{sub}$/mm	Repeated near-field scattering-center reconstruction and calibrated point geometry	0.230	0.282	0.386
$\Delta d_{res}$/mm	Independent geometric model-closure evaluation	0.306	0.367	0.459

**Table 5:** Pointwise mean residuals and repeatability statistics at 420 r/min.

$d_{R}$/mm	Mean d/mm	Mean Residual/mm	Repeatability SD/mm	CoV/%
99.000	99.415	0.415	0.180	0.181
198.000	196.882	−1.118	0.243	0.123
297.000	297.663	0.663	0.243	0.082
396.000	395.459	−0.541	0.329	0.083
495.000	496.310	1.310	0.218	0.044
594.000	593.627	−0.373	0.478	0.081
693.000	693.812	0.812	0.442	0.064
792.000	791.257	−0.743	0.544	0.069
891.000	892.868	1.868	0.482	0.054
990.000	988.532	−1.468	0.615	0.062

**Table 6:** Signed covariance-allocated contributions.

Uncertainty Group	Mean Contribution/%
Radar ranging	56.028
Vision-based construction angle	−0.020
Geometric registration	−0.290
Timing	0.001
Sensor-point substitution	21.159
Residual model discrepancy	23.121

**Table 7:** Sensitivity of the propagated uncertainty to the sensor-point substitution and residual model-closure components.

Scaling Factor	$u_{c}(d)$ Range/mm	Maximum Relative Change/%
0.8	0.637–0.913	−8.8
1.0	0.677–0.996	−
1.2	0.723–1.094	+9.8

**Table 8:** Comparison with representative dynamic blade-clearance measurement methods.

Method	Measurement Range	Sampling/Temporal Capability	Reported Accuracy or Uncertainty	Real-Time Capability	Environmental Characteristics
Airborne optical measurement, Bauknecht et al. [20]	Not specified as a ranging interval	1/rev, approximately 7.3 Hz	No unified output standard uncertainty reported	Phase-resolved in-flight measurement	Requires optical line of sight
Ultrasonic ranging, Zhang et al. [23]	100–1000 mm	Phase-triggered dynamic measurement	Maximum ranging error < 1.0%	Yes	Affected by acoustic propagation conditions
Coded ultrasonic ranging, Lu et al. [21,22]	100–1000 mm	Up to 3060 Hz at 1 m	Dynamic error within ±2 mm	Yes	Acoustic propagation remains environment dependent
Present radar–camera–encoder fusion method	99–990 mm	Event-triggered; validated at 300–600 r/min	$u_{c}(d)=0.677–0.996$ mm U(d)=1.354–1.993 mm	Yes, it meets the requirement of 300 to 600 r/min	Radar ranging is insensitive to illumination; the vision branch requires sufficient target visibility

## Data Availability

The original contributions presented in this study are included in the article. Further inquiries can be directed to the corresponding authors.
